# Boosting molecular diffusion following the generalized Murray's Law by constructing hierarchical zeolites for maximized catalytic activity

**DOI:** 10.1093/nsr/nwac236

**Published:** 2022-10-27

**Authors:** Ming-Hui Sun, Shu-Shu Gao, Zhi-Yi Hu, Tarek Barakat, Zhan Liu, Shen Yu, Jia-Min Lyu, Yu Li, Shu-Tao Xu, Li-Hua Chen, Bao-Lian Su

**Affiliations:** State Key Laboratory of Advanced Technology for Materials Synthesis and Processing, Wuhan University of Technology, Wuhan 430070, China; Laboratory of Inorganic Materials Chemistry (CMI), University of Namur, Namur B-5000, Belgium; Division of Analysis, Sinopec Beijing Research Institute of Chemical Industry, Beijing 100013, China; National Engineering Laboratory for Methanol to Olefins, Dalian National Laboratory for Clean Energy, Dalian Institute of Chemical Physics, Chinese Academy of Sciences, Dalian 116023, China; State Key Laboratory of Advanced Technology for Materials Synthesis and Processing, Wuhan University of Technology, Wuhan 430070, China; Nanostructure Research Centre, Wuhan University of Technology, Wuhan 430070, China; Laboratory of Inorganic Materials Chemistry (CMI), University of Namur, Namur B-5000, Belgium; State Key Laboratory of Advanced Technology for Materials Synthesis and Processing, Wuhan University of Technology, Wuhan 430070, China; State Key Laboratory of Advanced Technology for Materials Synthesis and Processing, Wuhan University of Technology, Wuhan 430070, China; State Key Laboratory of Advanced Technology for Materials Synthesis and Processing, Wuhan University of Technology, Wuhan 430070, China; State Key Laboratory of Advanced Technology for Materials Synthesis and Processing, Wuhan University of Technology, Wuhan 430070, China; National Engineering Laboratory for Methanol to Olefins, Dalian National Laboratory for Clean Energy, Dalian Institute of Chemical Physics, Chinese Academy of Sciences, Dalian 116023, China; State Key Laboratory of Advanced Technology for Materials Synthesis and Processing, Wuhan University of Technology, Wuhan 430070, China; State Key Laboratory of Advanced Technology for Materials Synthesis and Processing, Wuhan University of Technology, Wuhan 430070, China; Laboratory of Inorganic Materials Chemistry (CMI), University of Namur, Namur B-5000, Belgium

**Keywords:** zeolites, hierarchical Murray structure, ordered porous hierarchy, generalized Murray's Law, catalytic cracking

## Abstract

Diffusion is an extremely critical step in zeolite catalysis that determines the catalytic performance, in particular for the conversion of bulky molecules. Introducing interconnected mesopores and macropores into a single microporous zeolite with the rationalized pore size at each level is an effective strategy to suppress the diffusion limitations, but remains highly challenging due to the lack of rational design principles. Herein, we demonstrate the first example of boosting molecular diffusion by constructing hierarchical Murray zeolites with a highly ordered and fully interconnected macro–meso–microporous structure on the basis of the generalized Murray's Law. Such a hierarchical Murray zeolite with a refined quantitative relationship between the pore size at each length scale exhibited 9 and 5 times higher effective diffusion rates, leading to 2.5 and 1.5 times higher catalytic performance in the bulky 1,3,5-triisopropylbenzene cracking reaction than those of microporous ZSM-5 and ZSM-5 nanocrystals, respectively. The concept of hierarchical Murray zeolites with optimized structural features and their design principles could be applied to other catalytic reactions for maximized performance.

## Teaser

The first example of boosting molecular diffusion is reported by constructing hierarchical Murray zeolites with highly ordered and fully interconnected macro-meso-microporous structure following the generalized Murray's Law.

## INTRODUCTION

Diffusion, the decisive factor of mass transfer in microporous zeolite catalysts, is of crucial importance for their industrial utilization in catalysis, since the molecular mobility is a rate-limiting step of the overall catalysed reaction processes [1–3]. However, the diffusion of molecules in the micropores (the configurational diffusion) is at least one order of magnitude lower than those in mesopores (the Knudsen diffusion) and macropores (molecular diffusion) [4]. Such low diffusivities in micropores lead to restricted access and delayed transport of molecules to/from the catalytically active site constrained within micropores, resulting in short catalyst lifetime and poor zeolite utilization [5–7]. Introducing additional mesopores or/and macropores into the microporous zeolites offers an effective solution to enhance the mass transportation property for improved catalytic performance [2,5,8–20]. Kärger *et al*. prepared nanosheet assemblies of zeolite X with intracrystalline mesopores of 7 nm, which showed a cyclohexane diffusion enhancement by one order of magnitude and three times higher benzylchloride conversion in the benzylation of toluene compared to the purely microporous sample [12]. Pérez-Ramírez *et al*. synthesized mesoporous zeolite ZSM-5 by desilication, which exhibited a 6 times improved effective diffusivity of 2,2-dimethylbutane [13] and a 3.3 times extended cycle time in the conversion of methanol to olefin in comparison with traditional zeolites [14]. Our group reported an *in situ* bottom-up confined crystallization process to synthesize zeolite ZSM-5 single crystals with a fully interconnected and highly ordered intracrystalline macro–meso–microporosity [15]. Such hierarchically porous single crystal reactors provided a highly efficient diffusion system with the relative diffusion of bulky aromatic molecules 7 times higher and therefore a greatly improved catalytic performance with a lifetime 13 times longer than those of their microporous counterparts [15]. Most previously reported work on the synthesis of hierarchically porous zeolites lacks the rational design principles. Additionally, most of the aforementioned hierarchical pores are often disordered and not uniform although it is clear that the introduction of meso–macropores into microporous zeolites can more or less improve the catalytic performance. Further comprehensive work is needed to clarify how to optimize the hierarchically porous structure with predicted and rationalized pore size at each level for a maximized catalytic performance [21–25].

Pérez-Ramírez *et al.* proposed a hierarchy factor (HF) concept to design hierarchically porous zeolites [26], which describes the ratio between the relative mesoporous surface area (*S*_meso_/*S*_BET_) and the relative micropore volume (*V*_micro_/*V*_pore_). Higher HF refers to better matching of micro- and mesoporosity, and therefore leads to better catalytic performance. This HF concept focuses on the impact of micropores and mesopores. Further introduction of the effect of macropores together with the interconnection between different levels of pores and the quantitative analysis will offer the HF concept more power [26]. The evolution by natural selection creates various classes of hierarchically porous organisms, such as leaf veins, kidney, lung, vascular and respiratory systems for survival and production [2]. In 1926, Murray proposed the first law to stipulate the rational relationship between the pore radii ratio at each pore level. Murray's Law, using a biological consideration, is a general empirical principle of great utility in predicting porous parameters in bulk transport systems for connecting large vessels to small. In spite of strong interest, Murray's ideas went almost unnoticed for nearly a century, as this law is based on the assumption of constant flow [27]. Recently, our group revisited Murray's Law and established an equation (Supplementary Equation S1) that referred to the generalized Murray's Law, which predicts the precise diameter ratios for interconnected multi-scale pores from macroscopic to microscopic levels (Supplementary Equations S2 and S3), for the quantitative design of optimized hierarchically porous materials by taking the mass variations and constant surface substance exchange during mass transportations [28]. Following the generalized Murray's Law, hierarchically macro–meso–microporous ZnO materials (ZnO M–M–M) emulating the natural vascular structure were constructed and delivered a 2.5 times faster adsorption rate of rhodamine B than micro–mesoporous ZnO (ZnO M–M) in the photocatalytic degradation process [28]. With such Murray network architecture for fast mass diffusion and exchange in the liquid–solid reaction, ZnO M–M–M shows 2.5 times higher photocatalytic activity than that of ZnO M–M. Similarly, ZnO M–M–M also enables highly enhanced mass exchange and transfer in gas–solid and electrochemical reactions and exhibit enhanced 25- or 40-fold increases in performance compared to unimodal mesoporous materials performance in gas sensing and as Li-ion battery electrodes, respectively. This concept of ‘learning from nature’ offers inspiration in the quantitative design and synthesis of hierarchically porous materials with advanced performances. Regarding to the microporous structure of zeolites, the introduced macropores offer an unimpeded transport path while the introduced mesopores provide a fast pathway for mass transportation bridging the intrinsic micropores and the additional macropores [29]. Therefore, engineering the mesoporous and macroporous structure to match the zeolite micropores based on the generalized Murray's Law is essential to boost diffusion performances for maximized catalytic properties [30].

Herein, we demonstrate the proof of the concept of boosting molecular diffusion by constructing hierarchical Murray zeolites presenting a highly ordered and fully interconnected macro–meso–microporous structure whose pore-size ratio at each length scale is rationally modulated based on the generalized Murray's Law. For the first time, the generalized Murray's Law was used to establish the relationship between the structural characteristics, the diffusion behaviors and the catalytic performance in zeolite catalysis. Such a hierarchical Murray zeolite catalyst exhibits excellent mass transport properties with an effective diffusion rate of bulky 1,3,5-trimethylbenzene 9 and 5 times higher together with 2.5 and 1.5 times higher 1,3,5-triisopropylbenzene cracking conversions than purely microporous ZSM-5 and ZSM-5 nanocrystals, respectively. The strategy presented in this work enables the predictable and controlled synthesis of hierarchically porous zeolites with a quantitative hierarchy for optimized structural features and maximized performance.

## RESULTS AND DISCUSSION

**Figure 1.**
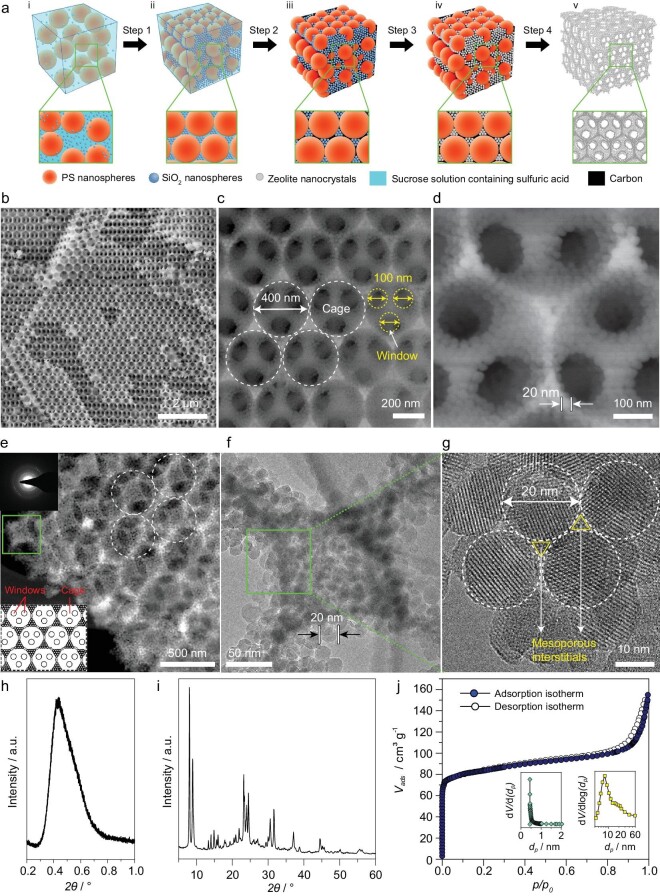
(a) Schematic of the synthesis of hierarchically ordered macro–meso–microporous zeolite ZSM-5 (OMMM–ZSM-5) assembled by zeolite nanocrystals. Steps: (1) self-assembly, (2) carbonization, (3) hydrothermal crystallization and (4) template removal by calcination. (i) Monodispersed polystyrene (PS) nanospheres and SiO_2_ nanospheres in sucrose solution, (ii) three-dimensionally periodic close-packed PS/SiO_2_ spheres in sucrose solution, (iii) PS/SiO_2_/carbon matrix, (iv) PS/ZSM-5 zeolite nanocrystals/carbon matrix and (v) OMMM–ZSM-5 by template removal via calcination. (b–j) Characterizations of OMMM–ZSM-5(400), as a representative sample. (b–d) SEM images. (e) TEM image and ED pattern (inset). (f) TEM image of enlarged area outlined in (e) by the green box. (g) HRTEM image of enlarged area outlined in (f). (h) SAXS and (i) WAXS pattern. (j) N_2_ adsorption–desorption isotherms and micropore-size, mesopore-size distribution (inset).

The confined crystalline transformation process was adapted to synthesize hierarchical Murray zeolite with a highly interconnected and three-dimensionally ordered macro–mesoporous structure assembled by uniform zeolite nanocrystals. The monodisperse polystyrene microspheres were used as templates for the three-dimensional ordered macropores. The walls of macropores are constructed from the assembly of highly uniform zeolite nanocrystals, which leads to the formation of an interconnected ordered mesoporous system. The interparticular mesopore size is determined by the size of the uniform zeolite nanocrystals. The detailed synthesis route is illustrated in Fig. 1a. First, the polystyrene spheres and the amorphous silica nanospheres (22 nm) were added into sucrose solution containing sulfuric acid to obtain stable dispersion (i in Fig. 1a). Three-dimensionally periodic close-packed polystyrene spheres/silica spheres (ii in Fig. 1a) were obtained via an evaporation-induced self-assembly method (Step 1 in Fig. 1a), followed by a preliminary carbonization process (Step 2 in Fig. 1a) to construct a PS/SiO_2_/carbon matrix. The PS/SiO_2_/carbon matrix acts as both the zeolite silica precursors and macropore/mesopore template. The *in situ*-formed carbon in the PS/SiO_2_/carbon matrix acted as a rigid supporting material to immobilize the three-dimensionally ordered close-packed PS nanospheres and SiO_2_ nanospheres and avoid the collapse of such an ordered structure during the crystalline process. Moreover, the confinement effect of the carbon ensures that the final size of the zeolite nanocrystals did not exceed the size of the original silica nanospheres (20 nm). This matrix was subsequently used as the zeolite silica precursors (iii in Fig. 1a) and was infiltrated with tetrapropylammonium ion (TPA^+^) and alumina source. TPA^+^ and alumina were permeated into the matrix evenly using a vacuum-rotary evaporation process. After being sealed in a Teflon-lined stainless-steel autoclave, the amorphous silica spheres located in the PS/SiO_2_/carbon matrix gradually reacted *in situ* with the aluminum precursor and crystallized around the structure directing agents TPA^+^ within the confined space during the hydrothermal process (Step 3 in Fig. 1a). The silica nanoparticles, located in the macropore walls and interstitials, transformed completely into zeolite crystals (iv in Fig. 1a). After template removal by calcination (Step 4 in Fig. 1a), the imprinted hierarchically ordered macro–meso–microporous structures constructed by zeolite nanocrystals (denoted as OMMM–ZSM-5(*x*), where OMMM represents the ordered macro–meso–microporous zeolite, and *x* represents the macropore size of the OMMM–ZSM-5 zeolites, *x* = 200, 400 and 600) were obtained (v in Fig. 1a).

Scanning electron microscope (SEM) images show that OMMM–ZSM-5(400), as a representative sample, has a highly ordered 3D inverse opal macroporous structure with an excellent regular periodicity (Fig. 1b). The ordered hexagonally arrayed cages of ∼400 nm are interconnected by windows of 100 nm (Fig. 1c and d). The walls shared by two adjacent macropores are completely constructed using uni-sized zeolite spherical nanocrystals of 20 nm (Fig. 1e and f) corresponding exactly to the size of the original amorphous SiO_2_ spheres used as the silica precursor. High-resolution (HR) transmission electron microscope (TEM) studies display that these nanospheres are pure zeolite ZSM-5 nanocrystals with high crystallinity and uniform particle size of 20 nm (Fig. 1f and g) in excellent agreement with the SEM observation. These monodisperse zeolite nanocrystals are densely packed to form ordered mesoporous interstitial voids (Fig. 1g). A strong diffraction peak can be found in the small-angle X-ray diffraction pattern (Fig. 1h), evidencing clearly the formation of the highly ordered mesostructure of the OMMM–ZSM-5(400). The wide-angle X-ray diffraction pattern of OMMM–ZSM-5(400) (Fig. 1i) further confirms the formation of a pure MFI zeolite phase with high crystallinity. The presence of micro- and mesopores in OMMM–ZSM-5(400) is further certified by nitrogen adsorption–desorption isotherms (Fig. 1j). OMMM–ZSM-5(400) shows a micropore-size distribution centered at 0.5 nm on the basis of the NLDFT (Nonlocal density functional theory) method, which is from the micropores of the ZSM-5 zeolite. The micropore surface area and volume of OMMM–ZSM-5(400) are 163 m^2^ g^−1^ and 0.09 cm^3^ g^−1^, respectively (Supplementary Table S1). The hysteresis loop observed in the isotherms corresponds to the abundant mesopores with the mesopore-size distribution centered at 8 nm. This pore size exactly corresponds to the interstitial diameter of the close-packed zeolite nanocrystals. The BET surface area and total pore volume of OMMM–ZSM-5(400) are 434 m^2^ g^−1^ and 0.3 cm^3^ g^−1^, respectively (Supplementary Table S1). The presence of meso- and macropores in OMMM–ZSM-5(400) is further illustrated by mercury intrusion porosimetry measurement (Supplementary Fig. S1). OMMM–ZSM-5(400) shows a mesopore distribution centered at ∼7.2 nm, which is almost the same as the value obtained by the N_2_ adsorption, and a narrow macroporous distribution centered at ∼100 nm corresponding to the window size of the macroporous cage. Due to the technique limit, the larger microporous cages of 400 nm observed using SEM (Fig. 1b–d) and TEM (Fig. 1e) are not seen using mercury intrusion porosimetry. The ^29^Si MAS NMR spectrum of OMMM–ZSM-5(400) shows a highly intense resonance at –114 ppm and a shoulder at –106 ppm, indicating that the framework consists primarily of cross-linked Q^4^ silica units [δ = –110, –114 and –118 ppm, Si(OSi)_4_] and Q^3^ silica units [δ = –102 nm, Si(OSi)_3_(OH)] (Supplementary Fig. S2a). The ^27^Al MAS NMR spectrum (Supplementary Fig. S2b) shows that aluminum atoms solely exist in the tetrahedral position (δ = 50 ppm) and no extra-framework aluminum species (δ = 0 ppm) are found, indicating that the amorphous aluminum atoms are all incorporated into the macro–meso–microporous framework in tetrahedral positions after the crystallization transformation process. The characterization data of OMMM–ZSM-5(200) and (600), conventional microsized (C–ZSM-5) and ZSM-5 nanocrystals (Nano–ZSM-5) are given in Supplementary Figs S3–S7).

The generalized Murray's Law (Supplementary Equation S1) was used to evaluate the hierarchically porous structural characteristics of our OMMM–ZSM-5 zeolites. The different size ratios between multi-scale pores can be expressed using Supplementary Equations S2 and S3 [28]. More insight into the establishment of these equations and the calculations of different pore sizes using these equations can be found in the supporting information. On the basis of the average number of micropores in one ZSM-5 spherical nanocrystal of our OMMM–ZSM-5 (Supplementary Equation S4 and Supplementary Fig. S8), when *D_mi__cro_* = 0.5 nm (Fig. 1j), the optimized diameter of the mesopores should be ∼10 nm in our OMMM–ZSM-5 (Supplementary Figs S9 and S10, and Supplementary Equation S2). The obtained mesopores of 8 nm (Fig. 1j) meet quite well the size ratios between micro- and mesopores. According to the generalized Murray's Law (Supplementary Equation S3 and Supplementary Fig. S10), the macropore size should be 440 nm. The experimental macropore size of the obtained OMMM–ZSM-5(400) is ∼400 nm (Fig. 1), which also very well matches the size ratios between macro- and mesopores. Two more OMMM catalysts with different macropore sizes (200 and 600 nm) were made for a better comparison. The obtained mesopores of 8 nm (Supplementary Figs S3d and S4d) meet quite well the size ratios between micro- and mesopores. The different size ratios between multi-scale pores can be expressed using Supplementary Equations S2 and S3 [28]. According to the generalized Murray's Law (Supplementary Equation S3 and Supplementary Figs S11 and S12), the macropore size should be 146 nm for OMMM–ZSM-5(200) and 812 nm for OMMM–ZSM-5(600). The experimental macropore sizes of the obtained OMMM–ZSM-5(200) and OMMM–ZSM-5(600) are ∼200 and ∼600 nm, respectively (Supplementary Figs S3 and S4), which fail to match the theoretically predicted and optimized size ratios between macro- and mesopores made by the generalized Murray's Law.

These results suggest that our OMMM–ZSM-5(400) obeys finely the pore-size ratio at micro-, meso- and macropore levels required by the generalized Murray's Law for optimized mass transport properties while OMMM–ZSM-5(200) and (600) fail to match exactly the meso–macropore-size ratio defined by the generalized Murray's Law. All the above results clearly demonstrate that hierarchical Murray zeolites with a high crystallinity and a highly interconnected, three-dimensionally ordered macro–meso–microporous structure assembled by uniform zeolite nanocrystals are obtained.

**Figure 2.**
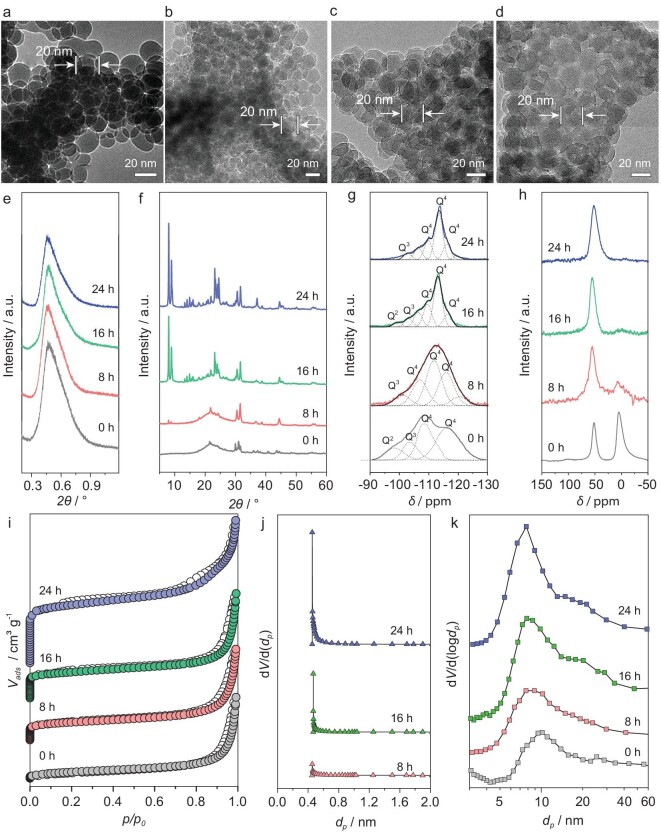
Crystallization process of OMMM–ZSM-5(400). TEM images of OMMM–ZSM-5(400) obtained at (a) 0 h, (b) 8 h, (c) 16 h and (d) 24 h. (e) SAXS data, (f) WAXS data, (g) ^29^Si NMR spectra (the black plots are the fitted data), (h) ^27^Al NMR spectra and (i) nitrogen adsorption isotherms, (j) micropore-size distributions and (k) mesopore-size distributions.

In order to gain deeper insight into the chemical crystalline transformation process for the formation of hierarchical Murray zeolites, samples at different crystallization times were collected and intensively characterized using a series of techniques. Using OMMM–ZSM-5(400) as a representative sample, SEM images (Supplementary Fig. S5) clearly show that the 3D highly ordered macroporous structure can be retained throughout the whole hydrothermal crystallization transformation process. TEM images display the same size of the monodisperse nanospheres in the samples with different crystallization times (Fig. 2a–d), suggesting that zeolite nanocrystals are derived from the amorphous silica nanospheres of the same size. Strong diffraction peaks are observed in the small-angle X-ray diffraction patterns for all the samples (Fig. 2e), indicating that the ordered mesoporous structure constructed from monodisperse nanospheres can be well retained during the crystallization process. The amorphous silica precursors gradually disappear and the crystallinity of the samples gradually increases, which is confirmed by the development of diffraction peaks corresponding to the zeolite MFI structure in the wide-angle XRD patterns (Fig. 2f). The XRD profile of OMMM–ZSM-5(400) reveals that a zeolite MFI structure can be obtained with a high degree of crystallinity after a crystallization transformation process of 24 h. These observations are further corroborated using ^29^Si MAS NMR spectroscopy to follow the transformation of the amorphous silica to zeolite nanocrystals during the crystallization process (Fig. 2g). As the reaction time increases, the intensity at both δ = –96 nm [Q^2^ groups, Si(OSi)_2_(OH)_2_] and δ = –102 nm [Q^3^ groups, Si(OSi)_3_(OH)] decreases while that at δ = –110, –114 and –118 ppm [Q^4^ groups, Si(OSi)_4_] increases, indicating the gradually increased crystallinity of the resultant zeolitic framework. Moreover, the resonance signals become narrower and no Q^2^ units exist in OMMM–ZSM-5(400), suggesting that the Si species are completely transformed from an amorphous to a crystalline framework after a hydrothermal reaction of 24 h. For ^27^Al MAS NMR spectroscopy (Fig. 2h), the signal centered at ∼0 ppm (octahedral Al) gradually decreases as the crystallization time extends, indicating that the aluminum atoms are progressively incorporated into the zeolitic framework at tetrahedral positions during the crystallization transformation process. Only one sharp, symmetrical signal centered at ∼50 ppm is observed in OMMM–ZSM-5(400), which means that the amorphous aluminum atoms in the precursor are all incorporated into the zeolitic framework at tetrahedral positions. N_2_ adsorption–desorption analysis of different samples further confirms the gradual generation of microporosity in the products (Fig. 2i and Supplementary Table S1). The surface area and pore volume of micropores increase gradually with increasing crystallization time (Supplementary Table S1). The micropore size of 0.5 nm calculated using the NLDFT method corresponds to the pore size of zeolite ZSM-5 (Fig. 2j). The mesopore-size distributions of samples collected at various crystalline times confirm that the mesopore size remains almost the same during the crystalline process (Fig. 2k). It can thus be concluded that the confined crystalline transformation process is a successful strategy to create hierarchical Murray zeolites with a highly interconnected, three-dimensionally ordered macro–mesoporous structure assembled by uniform zeolite nanocrystals.

**Figure 3.**
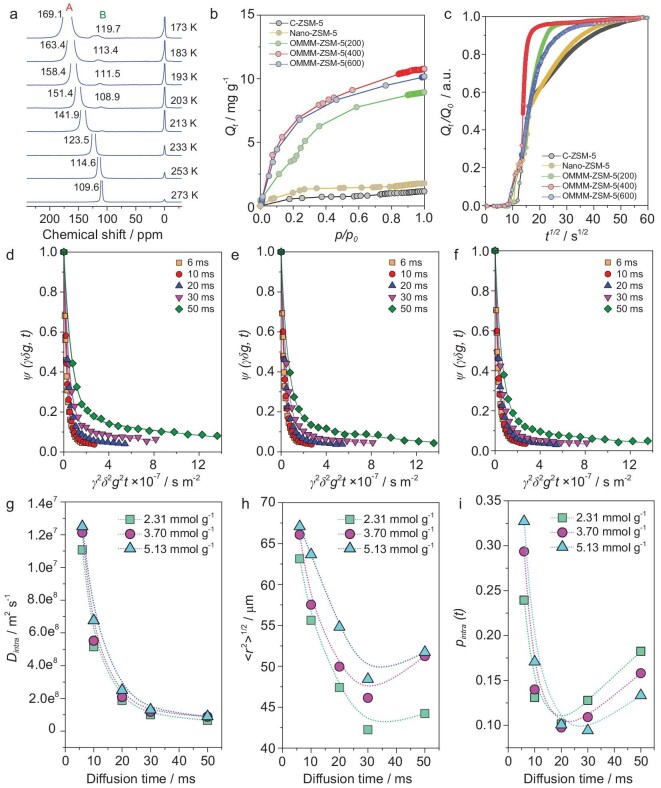
(a) Laser-hyperpolarized ^129^Xe NMR spectra with temperature varied from 273 to 173 K of OMMM–ZSM-5(400). (b and c) The macroscopic diffusion measurement. Adsorption and diffusion performance of 1,3,5-TMB within ZSM-5 samples. (b) The isothermal adsorption curve. (c) Normalized uptake (Q_t_/Q_0_) profiles of 1,3,5-trimethylbenzene over different catalysts. (d)–(h) The microscopic diffusion measurement. (d)–(f) ^1^H PFG NMR attenuation curves for methane with loading of (d) 2.31, (e) 3.7 and (f) 5.13 mmol·g^−1^ in OMMM–ZSM-5(400) for different observation/diffusion time *t* measures at 298 K. (g) Intracrystalline diffusivities *D_f__-intra_*, (h) the mean squared value of the displacements *〈 r*^2^ *〉* ^1/2^ and (i) the relative number of methane molecules still within OMMM–ZSM-5(400) *p_intra_*(*t*) of methane with different loadings in OMMM–ZSM-5(400) determined from PFG NMR attenuation curves in (d–f).

The interconnectivity between pores at different levels is essential for a hierarchical Murray structure and was first studied using the temperature-dependent hyperpolarized ^129^Xe nuclear magnetic resonance (HP ^129^Xe NMR, Fig. 3a). For comparison, the conventional microporous ZSM-5 (C–ZSM-5) with a similar Si/Al was used as the reference catalyst (Supplementary Fig. S6). A series of peaks at 0 ppm from 273 to 153 K are observed in C–ZSM-5 due to the presence of Xe in the gas phase at each temperature. Only one signal, line *A* (11–186 ppm), could be observed in the spectra of C–ZSM-5 from 273 to 153 K (Supplementary Fig. S13), which is ascribed to the Xe adsorbed in the 10-membered ring channel of ZSM-5 [15]. For OMMM–ZSM-5(400), the temperature-dependent behavior of line *A* in Fig. 3a is the same as that of C–ZSM-5 (Supplementary Fig. S13), indicating that the maintained microporosity in OMMM–ZSM-5(400). In addition to line *A*, a shoulder of *line B* appears ranging from 120 to 108 ppm at a low temperature from 203 to 173 K and the chemical shifts move downfield as temperature decreases. The shoulder and the new upfield *line B* at low temperatures are due to the Xe adsorbed in the mesopores and macropores in OMMM–ZSM-5(400). At a relatively high temperature of >203 K, the rapid exchange of Xe between the micropores and the macro–mesopores in the OMMM–ZSM-5(400) sample leads to the preferential adsorption of Xe in the micropores rather than in the mesopores and macropores, leading to the disappearance of the signals of *line B*. At high temperature, in addition to the signal corresponding to gas Xe, there is only one signal corresponding to the adsorption in micropores for OMMM–ZSM-5(400), suggesting that there is no other isolated pore and macro–mesoporosity is highly connected with intrinsic microporosity in OMMM–ZSM-5(400). These results very clearly indicate the excellent interconnectivity of the hierarchical Murray structure in OMMM–ZSM-5(400).

Concerning the mass transfer of bulky molecules within the highly interconnected hierarchical Murray structure of OMMM–ZSM-5, the intelligent gravimetric analysis (IGA), a macroscopic diffusion measurement, on the diffusion of 1,3,5-trimethylbenzene (1,3,5-TMB), was performed under inert conditions (Fig. 3b and c). For a better comparison, the conventional microporous ZSM-5 (C–ZSM-5, Supplementary Fig. S6) containing micro–mesopores and ZSM-5 nanocrystals (Nano–ZSM-5, Supplementary Fig. S7) containing micro–macropores were used as reference catalysts. It is clearly seen that OMMM–ZSM-5 zeolites have much larger max adsorption amounts at any relative pressure (Figs 3b and c) and a faster relative diffusion rate than those of C–ZSM-5 and Nano–ZSM-5 (Supplementary Table S2). This observation is directly linked to the excellent connectivity between the micropores and meso–macropores of OMMM–ZSM-5 zeolites. The highest adsorption amounts of 1,3,5-TMB and the highest diffusivity observed for OMMM–ZSM-5 (400) explain its optimized Murray structure. The above results show that the highly ordered and interconnected hierarchical macro–mesoporous structure inside our Murray zeolite is much more effective than micro–macroporous nanocrystals and the micro–mesoporous microsized conventional ZSM-5 zeolite. Such a unique hierarchically porous architecture can maximize the diffusion rate of reactants and products by reducing the effective diffusion length. The pore-size ratio obeying the generalized Murray's Law found in OMMM–ZSM-5(400) gives the best diffusion properties compared with the two other OMMM–ZSM-5(200) and (600) samples, which fail to match the theoretically predicted and optimized size ratios between macro- and mesopores defined by the generalized Murray's Law. However, these two samples still present much better diffusion performance than the C–ZSM-5 and Nano–ZSM-5 samples (Fig. 3b and c) showing the advantage of the introduction of a hierarchical micro–meso–macroporous structure into zeolites.

With regard to the intracrystalline diffusion behavior and the impact of the interconnected and rationalized macro–meso–micropores in accelerating diffusion in the OMMM–ZSM-5(400) zeolite, the ^1^H pulsed field-gradient (PFG) NMR, a microscopic diffusion measurement, was applied to OMMM–ZSM-5(400) as a representative sample. The methane, due to its small size, was chosen for these experiments with different loadings of 2.31, 3.70 and 5.13 mmol g^−1^ in OMMM–ZSM-5(400) zeolites over a wide range of diffusion times from 6 to 50 ms at 298 K. More description about the technique (Supplementary Equations S6–S12) can be found in the supporting information. The self-diffusivity *D_f_* is the slope in a semi-logarithmic plot of the PFG NMR signal attenuations versus the squared field-gradient pulse intensity (γδg)^2^. Two independent linear relationships are observed in all the plots (Fig. 3c–e). The first and steep decay corresponds to the methane molecules leaving the porous particles and diffusing rapidly in the interparticle space during the diffusion time *t*, as shown in the first part of Supplementary Equation S11. The second, more slowly decaying part of the attenuation represents the diffusion of methane strictly confined in the macro–meso–microporous structure during the diffusion time *t*, as shown in the second part of Supplementary Equation S11. Here we only consider the mass transfer within OMMM–ZSM-5(400), i.e. the intracrystalline diffusion. The intracrystalline self-diffusivities (*D_f__-intra_*) determined from the slope of the second part in the curves for different observation times *t* are shown in Fig. 3f. Compared to *D_f__-intra_* of methane molecules in purely microporous ZSM-5 in the order of 10^−9^ m^2^·s^−1^ [31], the values of *D_f__-intra_* of methane at different loadings in OMMM–ZSM-5(400) are in the order of 10^−8^ m^2^·s^−1^, which is 10 times higher, and this means that mesopores and macropores in OMMM–ZSM-5(400) can highly facilitate the diffusion of methane. The overall diffusion in OMMM–ZSM-5(400) is thus significantly improved. In Fig. 3g, we note that the mean squared value of the displacements *r*(*t*) significantly exceeds the size of the nanocrystals (∼20 nm), indicating that the transfer of the methane molecules through the OMMM–ZSM-5(400) hierarchical porous structure consists of displacements alternating between micro-, meso- and macropores. The total diffusivity of molecules (*D_f__-intra_*) in our hierarchical Murray zeolite is composed of the contribution from the diffusivity in micropores (*D_f__-micro_*), in mesopores (*D_f-meso_*) and in macropores (*D_f__-macro_*). Assuming that the mean lifetimes spent by the diffusing methane molecules within each type of pore is negligibly small compared with the observation time (fast-exchange condition [32]), Equation 1 can be obtained [33,34]:

(1) \begin{eqnarray*} {D}_{f \hbox{-} intra} &=& {p}_{micro} {D}_{f \hbox{-} micro} + {p}_{meso}{D}_{f \hbox{-} meso} \\ && +\, {p}_{macro}{D}_{f \hbox{-} macro}, \end{eqnarray*}

in which *p_x_* (*x* = micro, meso and macro, *p_micro_* + *p_meso_* + *p_macro_* = 1) is the relative number of molecules in the micro-, meso- and macropores, as defined on the basis of the Einstein relation (Supplementary Equation S8). The diffusivities in the meso- (*D_f-meso_*) and macropores (*D_f__-macro_*), offering many large mean free paths, belong to the Knudsen-type molecular propagation [33,35] and exceed the micropore diffusivities (*D_f__-micro_*) by at least one order of magnitude [36–38]. The *D_f__-intra_* in our OMMM–ZSM-5(400) obtained by ^1^H PFG NMR is ∼10 times higher than for a single microporous zeolite ZSM-5. This diffusion enhancement phenomenon can be directly attributed to the rational and quantitative relationship between macropores, mesopores and micropores in OMMM–ZSM-5(400), which obey the generalized Murray's Law, and their excellent interconnectivity. This is consistent with the HP ^129^Xe NMR and IGA results, indicating that such a unique hierarchical Murray architecture can boost the diffusion rate of reactants and products, leading to a much higher utilization efficiency of the active sites within zeolites.

The distribution of active sites is one of the most important factors to determine catalytic performance. To illustrate the extraordinary accessibility to active sites of OMMM–ZSM-5 zeolites, the acidic properties and the distribution of active sites were determined using ammonia temperature programmed desorption (NH_3_-TPD) analysis and the probe molecules [trimethylphosphine oxide (TMPO) and tributylphosphine oxide (TBPO)] adsorption method, respectively (Supplementary Table S3). The TMPO molecule with a kinetic diameter of 0.55 nm can penetrate into 10-membered-ring micropores (0.55 nm) and will give the total acidity of zeolites while the TBPO molecule is sterically bulky with a kinetic diameter of 0.82 nm and can only access the acid sites on the external surface out of micropores [39]. The acid sites constrained within the micropores are named as internal acid sites and all the acid sites out of micropores are external acid sites. The NH_3_-TPD profiles (Supplementary Fig. S14) indicate that the acidities in the three OMMM–ZSM-5(*x*) (*x* = 200, 400 and 600) are clearly similar to that in the two reference samples with the similar Si/Al ratios, C–ZSM-5 and Nano–ZSM-5 (Supplementary Table S3). This is because NH_3_ is a very small molecule with a molecule size of 0.26 nm and it can access to all the cages and channels of ZSM-5 zeolites. The NH_3_-TPD gives the total acidities of ZSM-5 zeolites. Their total acidities made using TMPO analysis are almost the same because the TMPO molecule with a kinetic diameter of 0.55 nm can penetrate into 10-membered-ring micropores (0.55 nm) and give the total acidity of zeolites. However, the external acidity made by a large TBPO molecule with a kinetic diameter of 0.82 nm is much higher for OMMM–ZSM-5(*x*) (*x* = 200, 400 and 600) (Supplementary Table S3). Nano–ZSM-5 has an improved adsorption capacity for TBPO (79 μmol·TBPO·g^−1^ cat) compared with that of C–ZSM-5 (41 μmol·TBPO·g^−1^ cat) due to its larger external surface area, while the OMMM–ZSM-5 sample contains much higher surface acidity. For example, the external surface acidity of OMMM–ZSM-5(400) (124 μmol·TBPO·g^−1^ cat) is three times higher than that of C–ZSM-5 and 30% higher than that of Nano–ZSM-5, respectively. These increased catalytic sites within the OMMM–ZSM-5 catalysts are due to their widely open framework, which is of great importance for catalytic performance.

The synergy of excellent diffusion properties and the abundant accessible acid sites makes hierarchical Murray zeolite ZSM-5 an extraordinary solid acid catalyst, especially for converting large organic molecules. The catalytic performances were evaluated in the cracking reaction of bulky 1,3,5-triisopropylbenzene (1,3,5-TIPB) to check the impact of improved diffusion properties. 1,3,5-TIPB molecules have larger kinetic molecular diameters (0.95 nm) than the pore openings of the zeolite ZSM-5 (0.55 nm) and thus are commonly used to study the external surface properties of different zeolites. The microsized ZSM-5 (C–ZSM-5) and the nanosized ZSM-5 (Nano–ZSM-5) have been used as reference catalysts. The pore-size distributions are added in Supplementary Figs S6c and S7c to give a clear understanding of the effectiveness of mesopores and macropores. The N_2_ adsorption–desorption isotherms (Supplementary Figs S6c and S7c) show clearly that the C–ZSM-5 has mesopores of 2 nm and the Nano–ZSM-5 has macropores of ∼50 nm. These two samples containing hierarchically multiple porosity (micro–mesopores for C–ZSM-5 and micro–macropores for Nano–ZSM-5) are thus excellent references for comparison with our micro–meso–macroporous Murray zeolites.

**Figure 4.**
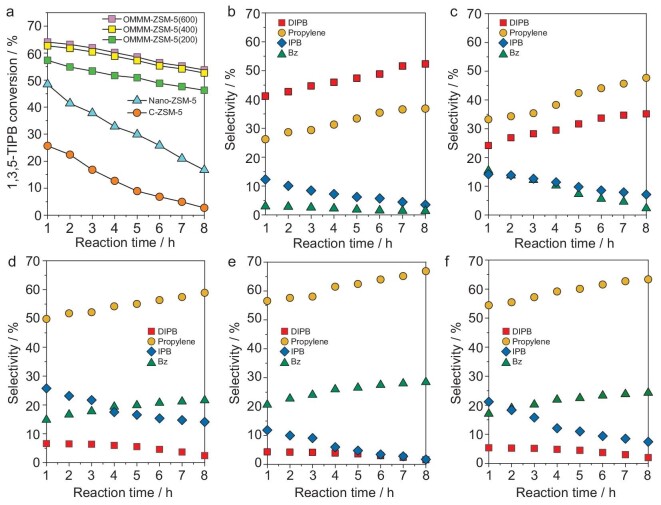
Catalytic performance of various zeolite ZSM-5 catalysts in the cracking reaction of 1,3,5-TIPB (a) catalytic activities (1,3,5-TIPB conversions) at different reaction times. (b–e) The product distributions in the 1,3,5-TIPB cracking using (b) C–ZSM-5, (c) Nano–ZSM-5, (d) OMMM–ZSM-5(200), (e) OMMM–ZSM-5(400) and (f) OMMM–ZSM-5(600).

The changes in both the 1,3,5-TIPB conversion and the associated product selectivity with time on stream are reported in Fig. 4. At each reaction time, C–ZSM-5 and Nano–ZSM-5 give very low and medium conversion, respectively. In contrast, OMMM–ZSM-5 catalysts are the most active. For more accurately estimating their catalytic activity, the turnover number (TON) values, normalized by the acidity amount of different catalysts, are shown in Supplementary Table S5 [40]. Clearly, the TON of all three OMMM–ZSM-5s at reaction time of 1–8 h are much higher than those of C–ZSM-5 and Nano–ZSM-5. This observation is directly linked to the excellent connectivity between micropores and meso–macropores of OMMM–ZSM-5 zeolites. Notably, for OMMM–ZSM-5(400), the TON value was 0.126 at a time on stream of 8 h, which is 10- and 3-fold higher compared to those of C–ZSM-5 and Nano–ZSM-5, respectively. At each reaction time, OMMM–ZSM-5(400) has the highest TON compared with that of OMMM–ZSM-5(200) and OMMM–ZSM-5(600). The highest TON observed for OMMM–ZSM-5 (400) is attributed to its optimized structure, which fully obeys the generalized Murray's Law. On the other hand, the product distribution provides information about the extent of the cracking degree. The deep cracking of 1,3,5-TIPB takes three successive steps, which are: first, dealkylation of 1,3,5-TIPB to form diisopropylbenzene (DIPB) and propylene; second, dealkylation of DIPB to give isopropylbenzene (IPB) and propylene; and third, dealkylation of IPB to give benzene (Bz) and propylene [41]. The main products catalysed by C–ZSM-5 are propylene and DIPB accompanied by few contents of IPB and benzene, indicating the low cracking degree of 1,3,5-TIPB. The DIPB content increases with the reaction time (Fig. 4b), suggesting the decreased cracking ability of C–ZSM-5. The interparticular mesopores in Nano–ZSM-5 offer better accessibility to external active sites and facilitate the diffusion of the bulky 1,3,5-TIPB as compared with C–ZSM-5 (Fig. 4c). The same decreasing trend of cracking ability with increasing reaction time is observed for Nano–ZSM-5. Significantly, the selectivity for the deep-cracking products over OMMM–ZSM-5 is much higher than those of C–ZSM-5 and Nano–ZSM-5 when compared at the same reaction time (Fig. 4d). The main products for OMMM–ZSM-5 are benzene and propylene with few contents of DIPB, indicating the much deeper cracking degree of 1,3,5-TIPB over OMMM–ZSM-5 catalysts. The selectivity for the deep-cracking products (propene and benzene) over OMMM–ZSM-5(400) with the optimized Murray structure is higher than those of OMMM–ZSM-5(200) and OMMM–ZSM-5(600) at the same reaction time (Fig. 4d). To compare the selectivity of the five catalysts at the equal conversion, the weight hourly space velocity was adjusted so as to obtain similar 1,3,5-TIPB conversions of ∼32% at 280°C, with the data shown in Supplementary Table S4. OMMM–ZSM-5(400) gave a very high selectivity for benzene product of 20.3% that is obviously higher than the other four catalysts. This result demonstrates that hierarchical Murray zeolites with an optimized structural feature can substantially promote the selectivity for deep-cracking products in the cracking of 1,3,5-TIPB. It is worth noting that although OMMM–ZSM-5(400) obeys exactly the generalized Murray's Law, OMMM–ZSM-5(200) and (600) still present much better catalytic properties than C–ZSM-5 and Nano–ZSM-5. Considering the similar total acidity of the three OMMM–ZSM-5(*x*) (*x* = 200, 400 and 600) and the two reference samples (C–ZSM-5 and Nano–ZSM-5), their high deep-cracking performances are attributed to the introduction of the fully interconnected hierarchically micro–meso–macroporous structures.

The deeper cracking process and higher catalytic stability of three OMMM–ZSM-5 catalysts than those of C–ZSM-5 and Nano–ZSM-5 should be attributed to their significant diffusion performance of bulky molecules and their low amount of coke deposition. The coke deposition on the reacted samples was analysed using thermogravimetric analysis (Supplementary Table S6). OMMM–ZSM-5(400) used in cracking of 1,3,5-TIPB for 8 h shows two times and 30% less coke deposition (6.7%) than C–ZSM-5 (13.3%) and Nano–ZSM-5 (9.4%). It is clear that the presence of an excellent hierarchical Murray structural diffusion system and strong accessible external surface acidity provides a highly efficient catalyst, exhibiting enhanced catalytic activity, deep-cracking ability as well as higher coke resistance in 1,3,5-TIPB catalytic cracking. Consequently, such a hierarchically ordered macro–meso–microporous Murray zeolite is a promising catalyst for many organic catalytic reactions involving large molecules.

In conclusion, the hierarchical Murray zeolite with highly ordered and fully interconnected macro–meso–microporous structure is successfully prepared via a hydrothermal chemical transformation process. Such a novel Murray structure has a rational and quantitative relationship between macropores, mesopores and micropores according to the generalized Murray's Law. The resultant hierarchical Murray zeolites exhibit excellent mass transport properties and thus consequently superior catalytic performance in the cracking of bulky 1,3,5-triisopropylbenzene. The generalized Murray's Law could enable predictable and controlled production of bio-inspired hierarchically porous materials with optimized structural features and highly enhanced performance.

## MATERIALS AND METHODS

### Synthesis of polystyrene spheres (∼420 nm)

In a typical synthesis, 47 g of styrene was added to 400 g of deionized H_2_O followed by adding 0.43 g of potassium persulfate. The reaction was performed at 80°C for 5 h in an argon atmosphere.

### Synthesis of hierarchically ordered polystyrene–silica–carbon composites

In a typical procedure [42], the as-synthesized polystyrene nanospheres were first blended with silica sol (LUDOX AS-40 colloidal silica, 40 wt% suspension in water, average particle size 22 nm, Sigma-Aldrich) under magnetic stirring for 1 h to obtain nanocomposite colloidal and then with sucrose at room temperature for 10 min, followed by the addition of sulfuric acid (95.0–98.0 wt% in water, sinoreagent) under stirring for another 10 min to obtain a stable dispersion. The typical mass ratio of the polystyrene nanospheres, silica nanospheres, sucrose and sulfuric acid was 100:15:15:1.5. The as-prepared dispersion was directly dried in an oven at 110°C for 6 h, then at 160°C for 6 h.

### Synthesis of hierarchically ordered macro–meso–microporous ZSM-5 nanocrystals

The polystyrene–silica–carbon composites were impregnated with an aqueous solution containing aluminum sodium oxide (NaAlO_2_, 99.99% metal basis, aladin) and tetrapropylammonium hydroxide (TPAOH, 1 M in water, aladin). Then the mixture was stirred for 1 h, transferred to a vacuum system and underwent rotary evaporation at 60°C to make sure there only existed a trace amount of water. The mixture was transferred to the autoclave under 130°C for a certain time. The products were washed using distilled water, dried in air at 60°C and finally calcined at 550°C for 7 h to remove the templates. The as-synthesized samples are denoted by OMMM–ZSM-5.

Commercial microporous ZSM-5 (C–ZSM-5) and ZSM-5 zeolite nanocrystals (Nano–ZSM-5) from FUYU New Materials Technology Co., Ltd were used as reference samples.

### Catalyst characterization

Small-angle X-ray scattering (SAXS) and wide-angle X-ray scattering measurements of solid samples were taken using a Bruker D8 Advance diffractometer with CuKα monochromatized radiation (λ = 1.5 418 Å).

SEM images were obtained using a Hitachi S4800 field-emission SEM operated at 5 kV and 10 μA. TEM images were performed using a Thermo Fisher Titan Themis 60–300 ‘cubed’ microscope fitted with double aberration-correctors, operated at 120 kV.

The chemical composition of the samples was determined using inductively coupled plasma optical emission spectroscopy (ICP–OES) using a PerkinElmer Optima 4300DV.

N_2_ adsorption–desorption isotherms were measured using a Micrometrics ASAP 2020 gas sorptometer after the samples were degassed at 573 K under a vacuum for 12 h.

The NMR spectra were recorded at room temperature using a Varian VNMRS spectrometer operating at 9.4 T (^27^Al freq.  = 79.46 MHz; ^29^Si freq = 79.46 MHz). The samples were packed in a standard 4-mm rotor and spun at 10 kHz. For ^27^Al, the parameters were: spectral width ∼104 kHz, relaxation delay 100 ms, excitation pulse 3 μs, acquisition time 5 ms. For ^29^Si, the parameters were: spectral width ∼104 kHz, relaxation delay 6 ms, excitation pulse 3 μs, acquisition time 5 ms.

Laser hyperpolarized ^129^Xe NMR experiments was used to investigate the interconnectivity between the macropore, mesopore and micropore. Both the 1,3,5-trimethylbenzene diffusion measurement in ZSM-5 zeolites, performed on a computer-controlled IGA (Hiden Analytical Ltd, Warrington, UK), a macroscopic diffusion measurement, and the ^1^H pulsed field-gradient (PFG) NMR, a microscopic diffusion measurement, were applied to investigate the intracrystalline diffusion and the impact of the interconnected macro–meso–micropores in accelerating the diffusion in zeolites. The details can be found in the supporting information.

The catalytic performances were evaluated in the cracking reaction of bulky 1,3,5-triisopropylbenzene (1,3,5-TIPB). The details can be found in the supporting information.

## Supplementary Material

nwac236_Supplemental_File
