# Supplementary material for: Boosting molecular diffusion following the generalized Murray's Law by constructing hierarchical zeolites for maximized catalytic activity
Source: Natl Sci Rev. 2022 Oct 27;9(12):nwac236. doi: 10.1093/nsr/nwac236 (PMC9828477; doi:10.1093/nsr/nwac236)
Supplement: nwac236_Supplemental_File [file nwac236_supplemental_file.docx]

Supporting Information

**Boosting molecular diffusion following the generalized Murray’s Law by constructing hierarchical zeolites for maximized catalytic activity**

Ming-Hui Sun^1,2,†^, Shu-Shu Gao^3,5,†^, Zhi-Yi Hu^1,4^, Tarek Barakat^2^, Zhan Liu,^1^ Shen Yu,^1^ Jia-Min Lyu,^1^ Yu Li,^1^ Shu-Tao Xu^5^, Li-Hua Chen^1,*^ and Bao-Lian Su^1,2,*^

^1^State Key Laboratory of Advanced Technology for Materials Synthesis and Processing, Wuhan University of Technology, Wuhan 430070, China;

^2^Laboratory of Inorganic Materials Chemistry (CMI), University of Namur, Namur B-5000, Belgium;

^3^Sinopec Beijing Research Institute of Chemical Industry, Beijing 100013, China;

^4^Nanostructure Research Centre, Wuhan University of Technology, Wuhan 430070, China;

^5^National Engineering Laboratory for Methanol to Olefins, Dalian National Laboratory for Clean Energy, Dalian Institute of Chemical Physics, Chinese Academy of Sciences, Dalian 116023, China

***Corresponding authors**. E-mails: chenlihua@whut.edu.cn; bao-lian.su@unamur.be

^†^Equally contributed to this work.

**Synthesis**

*Synthesis of uniform polystyrene (PS) spheres with tunable diameter*

The uniform polystyrene (PS) spheres with a diameter of 220 nm were synthesized by emulsion polymerization. 0.6 g Sodium laurylsulfonate (emulsifying agent) was dissolved in 360 g deionized H_2_O followed by addition of 47 g styrene. After adding 0.54 g potassium persulfate, the reaction was performed at 75 °C for 4.5 h under an argon atmosphere. Polystyrene spheres with monodispersed sizes of about 220 nm were obtained.

The uniform polystyrene (PS) spheres with a diameter of 420 nm were synthesized by soap-free polymerization. 47 g styrene were added in 400 g deionized H_2_O followed by addition of 0.43 g potassium persulfate. The reaction was performed at 80 °C for 5 h under an argon atmosphere. Polystyrene spheres with monodispersed sizes of about 420 nm were obtained.

The uniform polystyrene (PS) spheres with a diameter of 620 nm were synthesized by soap-free polymerization. 49 g styrene were added in 430 g deionized H_2_O followed by addition of 0.51 g potassium persulfate. The reaction was performed at 70 °C for 11 h under an argon atmosphere. Polystyrene spheres with monodispersed sizes of about 620 nm were obtained.

*Synthesis of Hierarchically ordered polystyrene-silica-carbon composites*

In situ self-assembly of colloidal polymer and silica spheres AS-40 (40 wt.% suspension in water, average particle size is 22nm nm, Sigma-Aldrich) with sucrose (ACS, aladin) as a carbon source was used to synthesize hierarchically ordered porous carbon with interconnected macropores and mesopores similar to the results of Zhang et al[1]. The typical mass ratio of polymer, silica, sucrose, and sulfuric acid was 100:15:15:1.5. In a typical procedure, polystyrene spheres were first blended with silica sol under magnetic stirring for 1 h to obtain nanocomposite colloidal and then with sucrose at room temperature for 10 min, followed by the addition of sulfuric acid (95.0-98.0 wt. % in water, sinoreagent) under stirring for another 10 min to obtain a stable dispersion. The as-prepared dispersion was directly dried in an oven at 110°C for 6 h, then at 160°C for 6 h.

*Synthesis of hierarchically ordered micro-meso-macroporous ZSM-5 nanocrystals*

Polystyrene-silica-carbon composites were impregnated with an aqueous solution containing Aluminum sodium oxide (NaAlO_2_, 99.99 % metal basis, aladin) and Tetrapropyl ammonium hydroxide (TPAOH, 1M in water, aladin). The ratio of n(Si): n(Al): n(TPA^+^) is 1: 0.02: 0.8 Then the mixture was stirred for 1h and transferred to the vacuum system and undergo rotary evaporation at 60 ℃ to make sure there only existed trace amount of water. The mixture was transferred to a Teflon-lined autoclave and heated at 130 ℃ for a certain time. The products were washed with distilled water, dried in air at 60 ℃, and finally calcined at 550℃ for 7 h to remove the polystyrene spheres and carbon matrix. The as-synthesized samples are denoted as OMMM-ZSM-5(x). OMMM represents ordered macro-meso-microporous zeolite, where x represents the macropore size of OMMM-ZSM-5 zeolites. With the size of PS sphere of 220 nm, 420 nm and 620 nm, OMMM-ZSM-5 samples with a macropore size of 200, 400 and 600 nm were obtained and labled as OMMM-ZSM-5(200), OMMM-ZSM-5(400) and OMMM-ZSM-5(600), respectively.

Commercial microsized ZSM-5 (C-ZSM-5) and nanosized ZSM-5 (Nano-ZSM-5) from FUYU New Materials Technology Co., Ltd. were used as reference samples with Si/Al ratios of 52 and 56, respectively.

**Catalyst Characterization**

Small-angle X-ray scattering (SAXS) and wide-angle X-ray scattering (WAXS) measurements of solid samples were taken on a Bruker D8 Advance diffractometer with CuKα monochromatized radiation (λ =1.5418 Å).

Scanning electron microscope (SEM) images were obtained on a Hitachi S4800 field-emission SEM operated at 5 kV and 10 μA. Transmission electron microscopy (TEM), high resolution transmission electron microscopy (HR-TEM), selected area electron diffraction (SAED) and high angle annular dark field scanning transmission electron microscope (HAADF-STEM) were performed on a Thermo Fisher Titan Themis 60-300 cubed microscope fitted with double aberration-correctors for both TEM and STEM, operated at 120 kV.

The chemical composition of the samples was determined by inductively coupled plasma optical emission spectroscopy (ICP-OES) using a PerkinElmer Optima 4300DV. The wavelength range was 165~782nm and resolution was 0.006nm (at 200nm).

N_2_ adsorption-desorption isotherms were measured using a Micrometrics ASAP 2020 gas sorptometer after the samples were degassed at 573K under vacuum for 12h. The micropore surface area were determined from N_2_ adsorption using t-plot method. By using NLDFT modal, micropore volumn and micropore size were derived from the adsorption branches of N_2_ isotherms when the relative pressure p/p_0_ under 0.01. The mesopore surface area were determined from N_2_ adsorption using BET equation. Total pore volumes were estimated from the adsorbed amount at a relative pressure p/p_0_ of 0.99. By using BJH modal, mesopore size was determined by the adsorption branches of N_2_ isotherms.

The MAS spectra were recorded at room temperature, using a Varian VNMRS spectrometer operating at 9.4 T (^27^Al freq. = 79.46 MHz; ^29^Si freq = 79.46 MHz). The used probe was a Varian/Chemagnetics HX 4 mm CPMAS. The samples were packed in a standard 4mm rotor and spun at 10 kHz. The number of transients ranges between about 200 and 11000 for the ^29^Si spectra, and between 2000 and 3500 for the ^27^Al spectra. Specially for ^27^Al, the parameters were: spectral width about 104 kHz, relaxation delay 100 ms, excitation pulse 3 us, acquisition time 5 ms. For ^29^Si, the parameters were: spectral width of about 104 kHz, relaxation delay 6 ms, excitation pulse 3 us, acquisition time 5 ms.

Laser hyperpolarized ^129^Xe NMR experiments were carried out at 110.6 MHz on a Varian Infinity-plus 400 spectrometer using a 7.5 mm probe. Before each experiment, samples (60–80 mesh) were dehydrated at 673 K under vacuum (<10^-5^ Torr) for 24 h. The optical polarization of xenon was achieved with a homemade apparatus with the optical pumping cell in the fringe field of the spectrometer magnet and a 60 W diode laser array (Coherent FAP-System). A flow of gas mixture (1% Xe – 1% N_2_ – 98% He) was delivered at the rate of 100–150 mL min^-1^ to the sample in the detection region via plastic tubing. Variable-temperature NMR measurements were performed in the range of 153–273 K. All one-dimensional spectra were acquired with 3.0 μs π/2 pulse, 100–200 scans, and 2 s recycle delay. The chemical shifts were referenced to the signal of xenon gas. Although the line of the xenon gas is temperature dependent, generally chemical shifts vary no more than 1 ppm in the temperature range of the experiments.

***Acidity measurement:*** ***NH_3_ temperature-programmed desorption (NH_3_ TPD)***

The acidity of the samples was determined by ammonia NH_3_ temperature programmed desorption using a ChemBET Pulsar. For measurement, 120 mg powder sample was introduced into a quartz reactor and degassed under He flow at 600 °C. After cooling to room temperature, NH_3_ gas was adsorbed for 1 h after which the physisorbed NH_3_ was removed under He flow at 50°C. Desorption profile was then measured with evacuation at the temperature gradient of 10 °C min^-1^, using a TCD detector.

***Quantitative investigation of the internal and external acidity***

The internal and external acidity of zeolite sample was determined by the adsorption of trimethylphosphine oxide (TMPO, 100%, Alfa) and tributylphosphine oxide (TBPO, 98%, Acros) method[2]. In a typical procedure, the dehydrated samples were added into the TMPO/anhydrous CH_2_Cl_2_ solution or TBPO/anhydrous CH_2_Cl_2_ solution in a N_2_ glovebox. To ensure uniform adsorption of the probe molecules on the samples, the loaded sample was agitated in an ultrasonic shaker for 1 h after thorough mixing and then sit for 12 h. To remove the CH_2_Cl_2_ solvent was achieved, the samples were extracted under liquid N_2_ and then evacuated at 50°C. ICP-OES spectroscopy was performed to determine the total P content of the zeolite after being titrated by TMPO or TBPO. Typically, 100 mg of the adsorbate loaded sample was dissolved in 10 mL of a HF, HNO_3_, and HCl mixture and then added to 60 mL of a saturated boric acid solution at room temperature. The concentration of P, Si and Al elements were determined using commercial standards (High-purity standards). Three scans were obtained for each element (Si, Al, P) and averaged to determine the concentration of each element from established calibration curves.

**Catalytic activity test**

Before catalytic reactions, the zeolites were firstly ion-exchanged with 1M NH_4_NO_3_ solution for three times at 80°C for 6h and then converted into H^+^-form by calcination in air at 550°C for 6 h. Typically, the as-synthesized H^+^-form zeolite catalysts were degassed at 300°C for 12h. The gas-phase cracking of 1,3,5-TIPB was performed to evaluate the catalytic property of the zeolite catalysts for bulky molecules processing. The 1,3,5-TIPB cracking reactions were carried out using a continuous flow system in a fix-bed quartz reactor under atmospheric pressure using nitrogen as a carrier gas. 0.5 g of the zeolite catalyst, diluted with 2.0 g of quartz beads was placed in the reactor. Before the reaction, the catalyst was activated at 500°C in nitrogen flow (30 mL min^-1^) for 2 h. The reactor temperature was then decreased to the reaction temperature (300°C)and the liquid reactant 1,3,5-TIPB was fed with a flow of 1.5g h^-1^ , i.e., weight hourly space velocity (WHSV) of 3 h^-1^. The liquid products were collected periodically and analyzed on a gas chromatography (Agilent 7890B, FID detector).


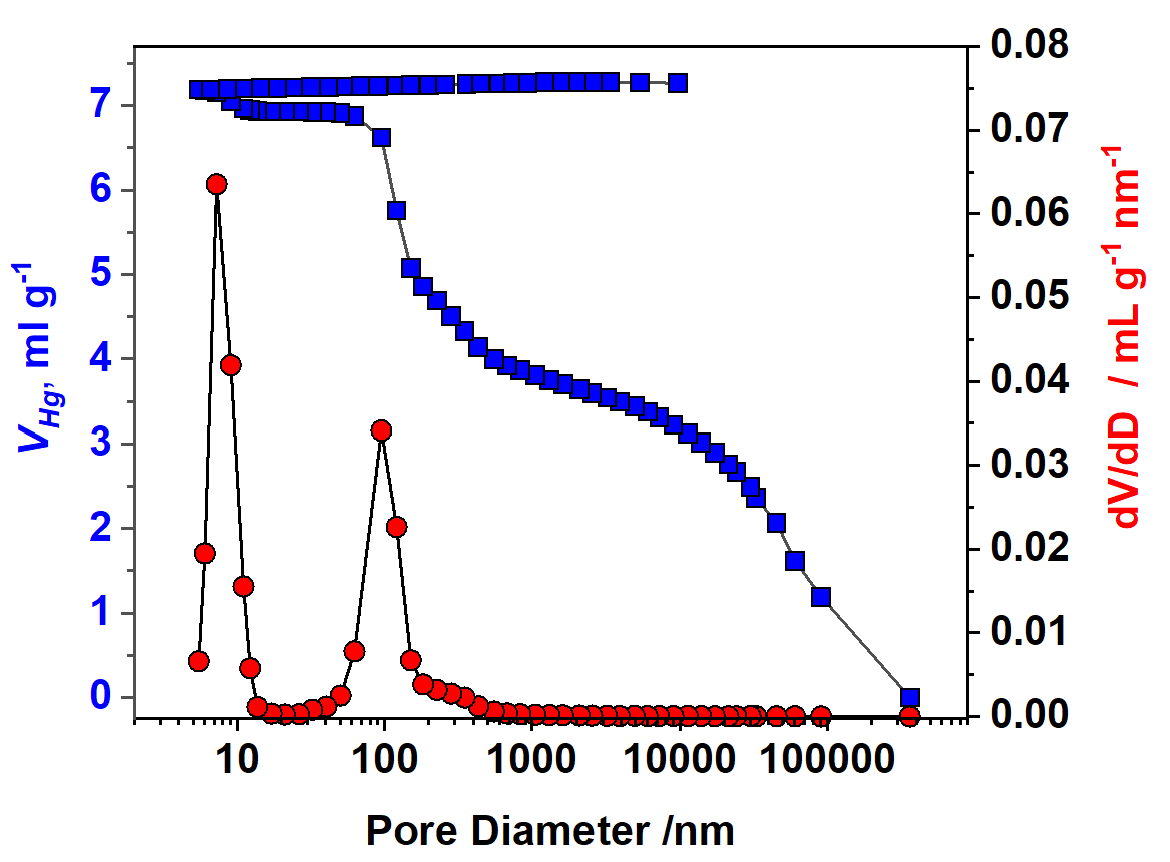


**Figure S1** Mercury intrusion curves of OMMM-ZSM-5(400).


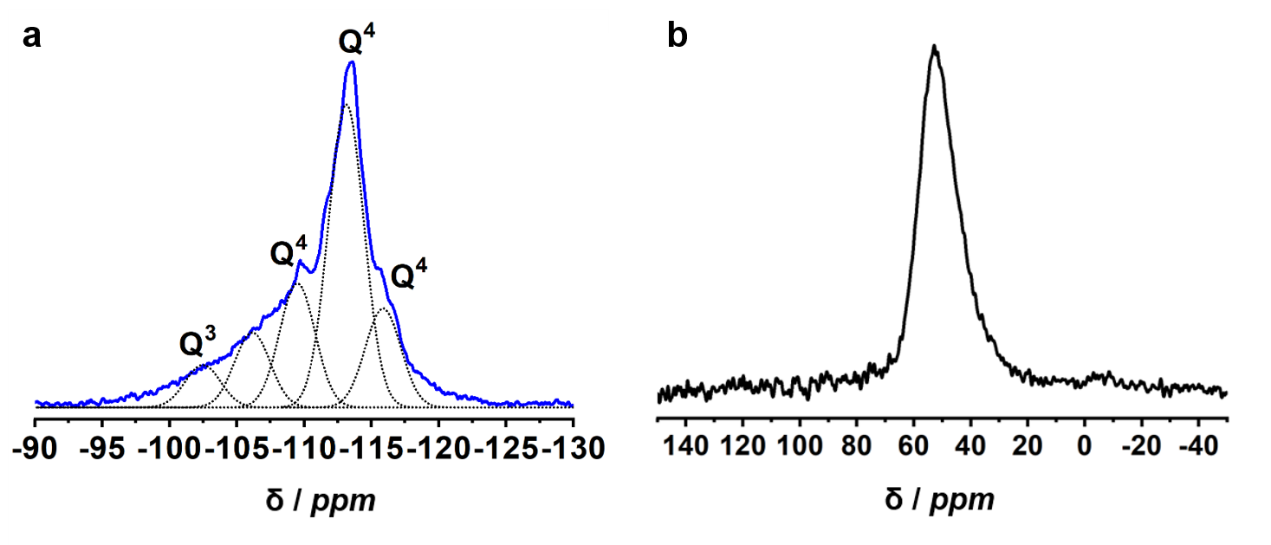


**Figure S2** (a)^29^Si MAS NMR and (b) ^27^Al MAS NMR spectra of OMMM-ZSM-5(400).


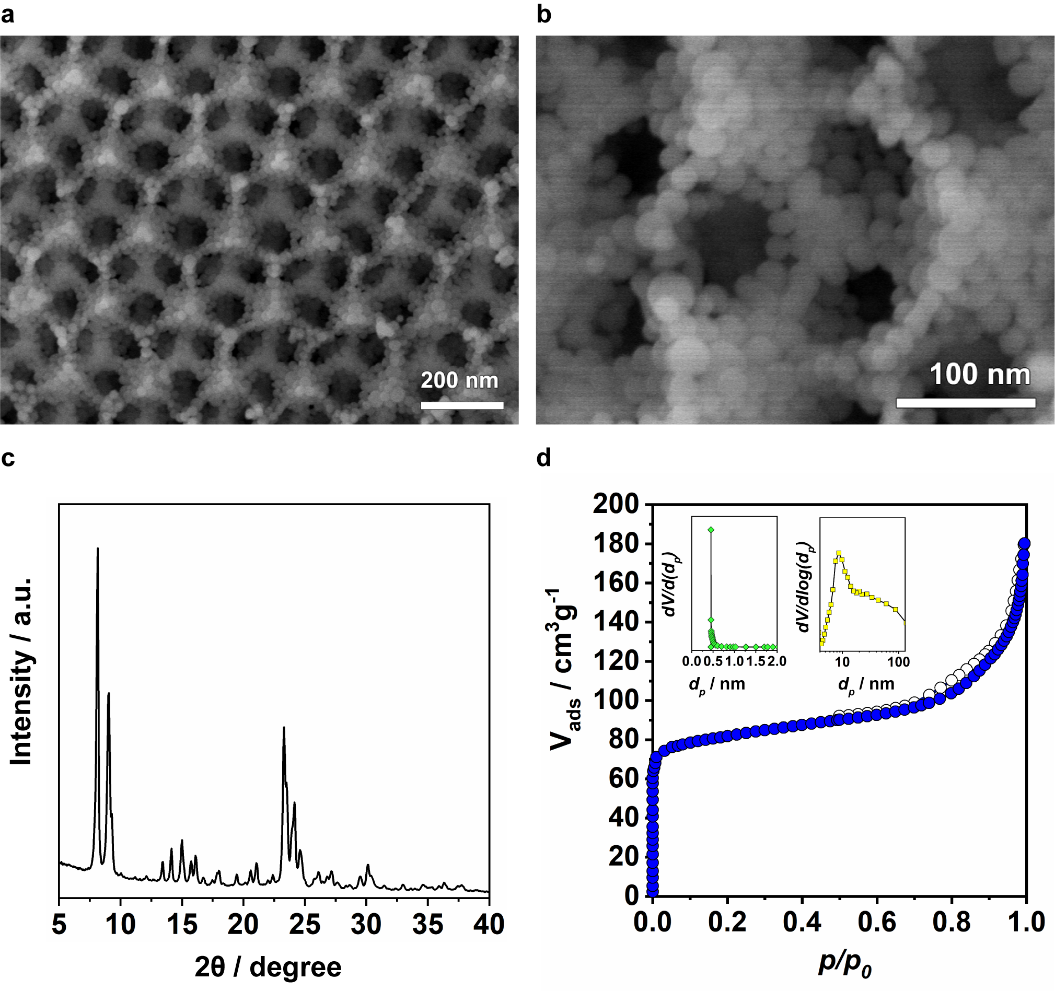


**Figure S3** (a, b) SEM images, (c) XRD patterns and (d) N_2_ adsorption-desorption isotherms and micropore-size, mesopore-size distribution (inset) of OMMM-ZSM-5(200).


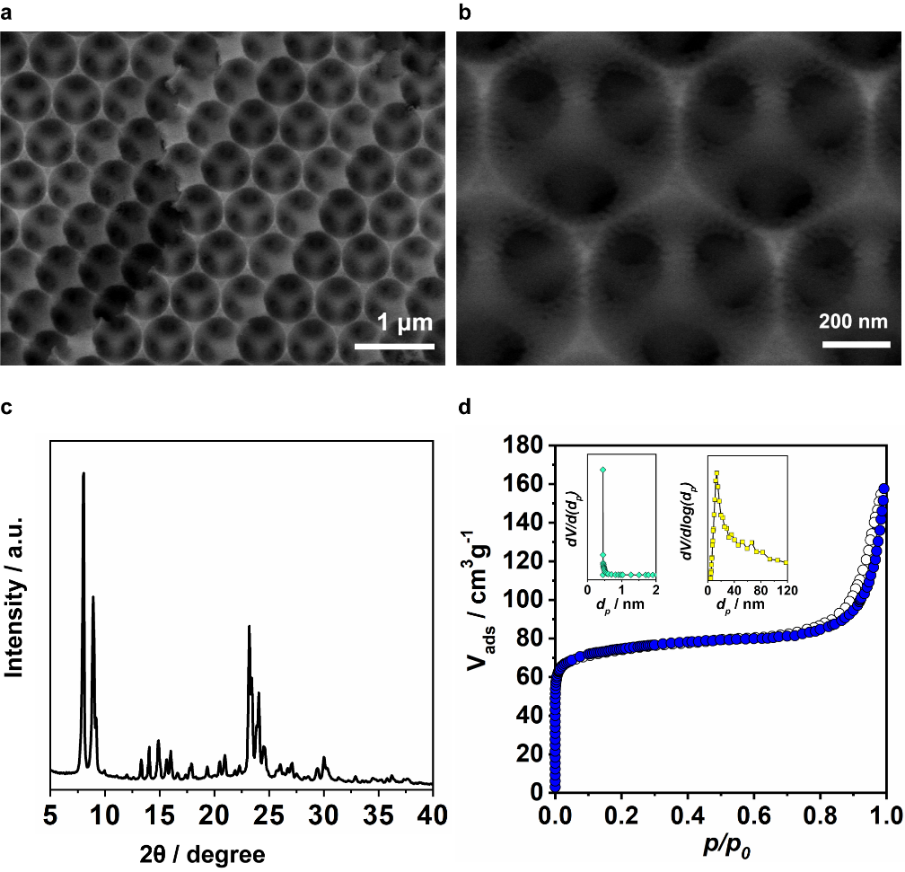


**Figure S4** (a, b) SEM images, (c) XRD patterns and (d) N_2_ adsorption-desorption isotherm and micropore-size, mesopore-size distribution (inset) of OMMM-ZSM-5(600).

**
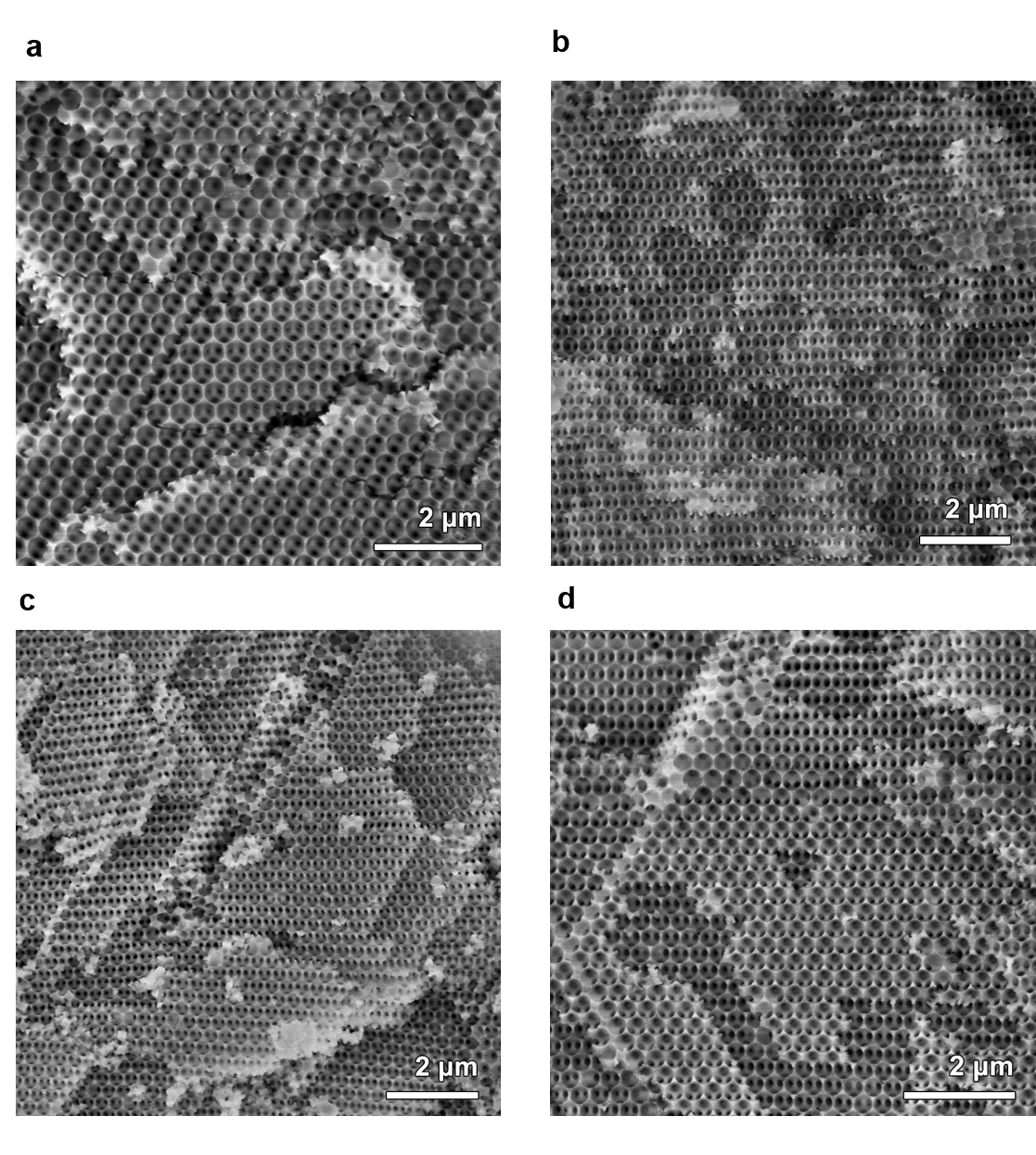
**

**Figure S5** Crystallization process of OMMM-ZSM-5(400). SEM images of OMMM-ZSM-5(400) obtained at (a) 0h, (b) 8h, (c) 16h and (d) 24h.


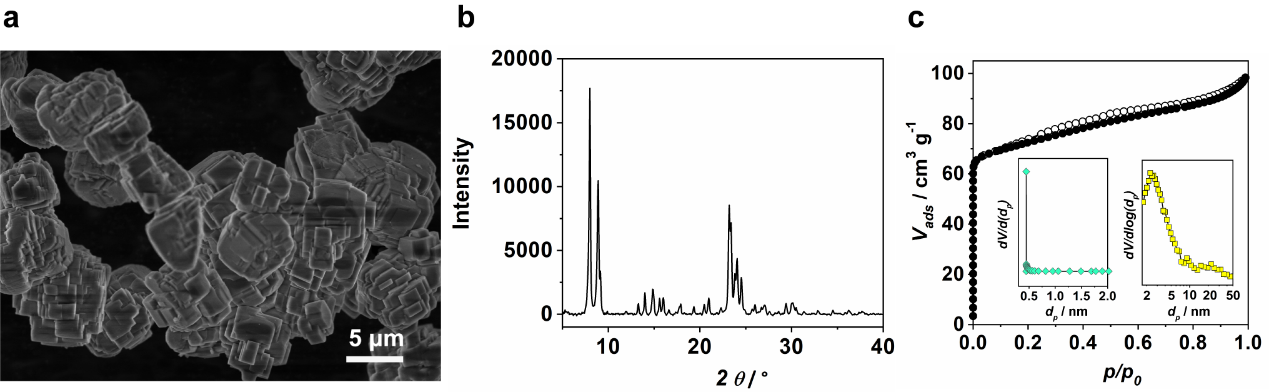


**Figure S6** (a) SEM image, (b) XRD, (c) N_2_ adsorption-desorption isotherm and micropore-size, mesopore-size distribution (inset) of C-ZSM-5


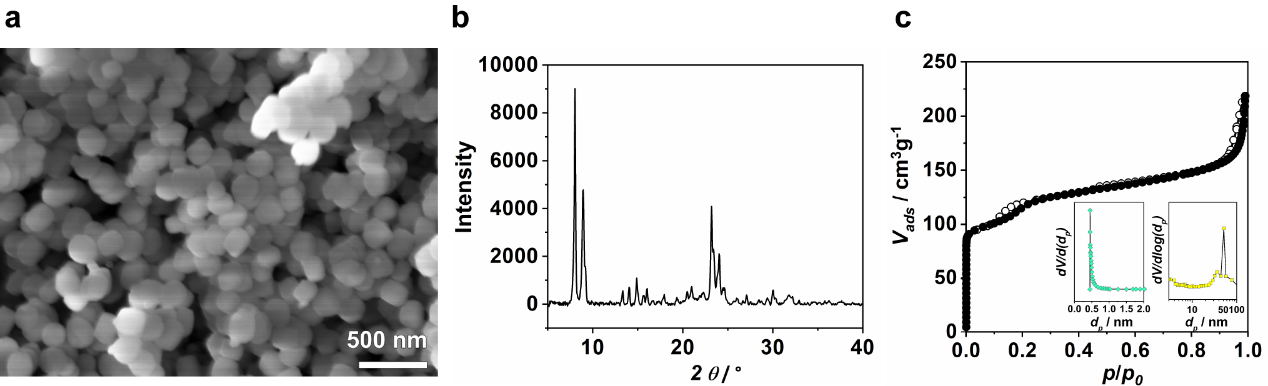


**Figure S7** (a) SEM image, (b) XRD, (c) N_2_ adsorption-desorption isotherm and micropore-size, mesopore-size distribution (inset) of Nano-ZSM-5

OMMM-ZSM-5(400) showed combined features of type I and type IV isotherms with two steep steps in the p/p_0_ < 0.01 and 0.60 < p/p_0_ < 1.0 regions, corresponding to filling of the micropore volumes and capillary condensation in the mesopores, respectively[3]. It is noteworthy that the isotherms also have an abrupt climb at the pressure of p/p_0_ ≈ 1, characteristic of the meso-macroporous materials[4]. The H1-type hysteresis loop of the isotherm suggests that the mesopores were interconnected through large openings without restriction on the capillary evaporation of the adsorbed gas[3]. The BET surface area and total volume are 434 m^2^g^−1^ and 0.30 cm^3^g^−1^ for OMMM-ZSM-5(400) (Table S1). The micropore size distribution determined from N_2_ adsorption using the NLDFT model is centered at ~0.5 nm (insets of Figures 1j) for OMMM-ZSM-5(400). The micropore surface area and volume are 163 m^2^g^−1^ and 0.09 cm^3^g^−1^ for OMMM-ZSM-5(400) (Table S1).

Two more OMMM catalysts with different macropore sizes (200nm and 600nm) were made for a better comparison. The size of the macropores in OMMM zeolites can be tuned by varying the size of the corresponding polystyrene spheres (220 nm and 620 nm, respectively). The as-synthesized samples are denoted by OMMM-ZSM-5(*x*). OMMM represents ordered macro-meso-microporous zeolite, where *x* represents the macropore size of OMMM-ZSM-5 zeolites. Figures S3 and S4 show the structural and textural characterizations of OMMM-ZSM-5(200) and OMMM-ZSM-5(600), respectively. SEM images of OMMM-ZSM-5(200) (Figure S3a,b) and OMMM-ZSM-5(600) (Figure S4a,b) show that after 24 h of crystallization, these two samples both have hierarchically ordered macro-mesoporous structure (~200 nm and ~600 nm, respectively), assembled by uniform small zeolite nanospheres (~20 nm) and are further characterized by XRD showing highly crystalline MFI-type zeolite patterns (Figures S3c and S4c, respectively).

OMMM-ZSM-5(200) and OMMM-ZSM-5(600) both showed combined features of type I and type IV isotherms (Figures S3d and S4d) with two steep steps in the p/p_0_ < 0.01 and 0.60 <p/p_0_ <1.0 regions, corresponding to filling of the micropore volumes and capillary condensation in the mesopores, respectively[3]. It is noteworthy that the isotherms also have an abrupt climb at the pressure of p/p_0_ ≈ 1, characteristic of the meso-macroporous materials[4]. The H1-type hysteresis loop of the isotherms suggest that the mesopores were interconnected through large openings without restriction on the capillary evaporation of the adsorbed gas[3]. The BET surface area and total volume are 417 m^2^g^−1^ and 0.28 cm^3^g^−1^ for OMMM-ZSM-5(200) and 428 m^2^g^−1^ and 0.25 cm^3^g^−1^ for OMMM-ZSM-5(600), respectively (Table S1). The micropore size distributions determined from N_2_ adsorption using the NLDFT model both are centered at ~0.5 nm (insets of Figures S3d and S4d, respectively) for OMMM-ZSM-5(200), OMMM-ZSM-5(400) and OMMM-ZSM-5(600). The micropore surface area and volume are 170 m^2^g^−1^ and 0.09 cm^3^g^−1^ for OMMM-ZSM-5(200) and 185 m^2^g^-1^ and 0.09 cm^3^ g^-1^ for OMMM-ZSM-5(600), respectively (Table S1). Such high values in external surface area and total pore volume are owing to the significant amount of mesopores.

In contrast, C-ZSM-5 showed type I isotherms with one steep steps in the p/p_0_ < 0.01, corresponding to filling of the micropore volumes and another step in the 0.20 < p/p0 < 0.80, corresponding to the capillary condensation in the mesopores (Figure S6c). The micropore surface area and micropore volume are 153 m^2^g^−1^ and 0.08 cm^3^g^−1^ respectively (Table S1). The BET surface area and total volume are 220 m^2^g^−1^ and 0.15 cm^3^g^−1^ (Table S1). BJH method gives a pore size distribution around 2 nm, which are formed by desilication during the crystallization process. N_2_ sorption isotherm of OMMM-ZSM-5(400) (Figure 1j) shows a nitrogen adsorption capacity around 155 cm^3^g^−1^ which is much higher than that for C-ZSM-5 (98 cm^3^g^−1^). This is due to the presence of abundant macro-mesopores within OMMM-ZSM-5(400).

Nano-ZSM-5 exhibited type I adsorption-desorption isotherms (Figure S7c) with one steep steps in the p/p0 < 0.01, corresponding to filling of the micropore volumes and another step in the 0.9 < p/p0 < 1.0, corresponding to existence of macropores (Figure S7c). The micropore surface area and micropore volume are 81 m^2^g^−1^ and 0.03 cm^3^g^−1^ respectively (Table S1). The BET surface area and total volume are 385 m^2^g^−1^ and 0.29 cm^3^g^−1^ (Table S1). BJH method gives a pore size distribution around 51 nm. These macropores are generated by the aggregation of nanocrystals of Nano-ZSM-5. This part of results shows clearly that both the C-ZSM-5 and theNano-ZSM-5 contain multiple porosity (micro-mesopores for C-ZSM-5 and micro-macropores for Nano-ZSM-5), but without interconnection.

**Design of Murray zeolites following the generalized Murray’s law**

The generalized Murray’s Law established on the basis of original Murray’s Law predicts the precise diameter ratios for interconnected multi-scale pores from macroscopic to microscopic levels of hierarchically porous systems for minimized transport resistance[5]. It indicates that macropore size decreases along different scales and ends in units of constant size. The generalized Murray’s Law takes the mass variation and constant surface substance exchange during the mass transportation (Equation S1)[5].

${\boldsymbol{\gamma}_{\boldsymbol{0}}}^{\boldsymbol{\alpha}}\boldsymbol{=}\frac{\boldsymbol{1}}{\boldsymbol{1-X}}\sum_{\boldsymbol{i=1}}^{\boldsymbol{N}} {\boldsymbol{\gamma}_{\boldsymbol{i}}}^{\boldsymbol{\alpha}}$ (Equation S1)

where the exponent *α* (2 or 3) is dependent on the type of the transfer, for hierarchical pores, *r_0_* is the radius of a parent pore and *r_i_* is radius of children pores and *X* is the ratio of mass variation during mass transfer in the parent pore. This demands a reasonable tolerance adjusted for area-preserving branching networks involving mass diffusion or ion transfer (*α = 2*) with mass variations during the optimum transfer process.

For a two-dimensional hierarchical Murray material, the generalized Murray’s Law can be expressed by the following equations:

${\boldsymbol{D}_{\boldsymbol{meso}}}^{\boldsymbol{2}}\boldsymbol{=}\frac{\boldsymbol{1}}{\boldsymbol{1-X}}\sum{\boldsymbol{D}_{\boldsymbol{micro}}}^{\boldsymbol{2}}\boldsymbol{=}\frac{\boldsymbol{1}}{\boldsymbol{1-X}}\frac{\boldsymbol{1}}{\boldsymbol{d}}\boldsymbol{nl}{\boldsymbol{D}_{\boldsymbol{micro}}}^{\boldsymbol{2}}$ (Equation S2)

where $1-X=\frac{S_{micro}}{S}$

$D_{macro}=\pi h\frac{{D_{meso}}^{2}}{d^{2}}$ (Equation S3)

The physical relationships in the equations can be used as a guide for the fabrication of Murray materials. These equations reveal that the micro-scale parameters for materials, including the film thickness (*h*), wall width (*l*) and diameter (*D_macro_*) of the macropore are closely dependent on the nanoscale structural parameters of the NP building-blocks (*d*, *n*, *S*, *S_micro_*, *D_micro_* and *D_meso_*). Here, *d* is the diameter of nanoparticles, *n* is the average number of micropores within a single NP, *S* is the specific surface area of all the NPs, *S_micro_* is the surface area of the micropores *D_micro_* and *D_meso_* are the diameters of micropores and mesopores, respectively.

We use the generalized Murray’s law to design and optimize the structures of three-dimensional hierarchically porous materials[5]. Figure 1a shows the pore model abstracted from our hierarchically macro-meso-microporous catalysts self-assembled by zeolite ZSM-5 nanocrystals. To connect macropores with mesopores, and mesopores with micropores, the size ratios between multi-scale pores are derived based on the generalized Murray’s law.

In the present case, *D_micro_*, *D_meso_* and *D_macro_* are the diameters of the micropores, mesopores and macropores, respectively. *X* is the the ratio of mass variation during mass transfer in the parent pore. *S_micro_* is the microporous specific surface area (*S_micro_*= 163 m^2^g^-1^, Table S1) and *S* is the total specific surface area (*S*=*S_BET_*=434 m^2^g^-1^, Table S1). *d* is the diameter of zeolite nanocrystals (*d*=20 nm, Figure 2g-2g). *n* is the average number of the micropores within one zeolite nanocrystal. *l* is the length of the mesopores, equating to the average height of the octahedron surrounded by six adjacent zeolite nanocrystals. *h* is the diffusion distance from one macropore to another through meso-microporous wall. The average number of the micropores within one zeolite nanocrystal *n* is calculated as follows:

The ZSM-5 zeolites have a structural code “MFI” by International Zeolite Association (IZA) database. The microporous network of this zeolite has intersecting straight and zigzag


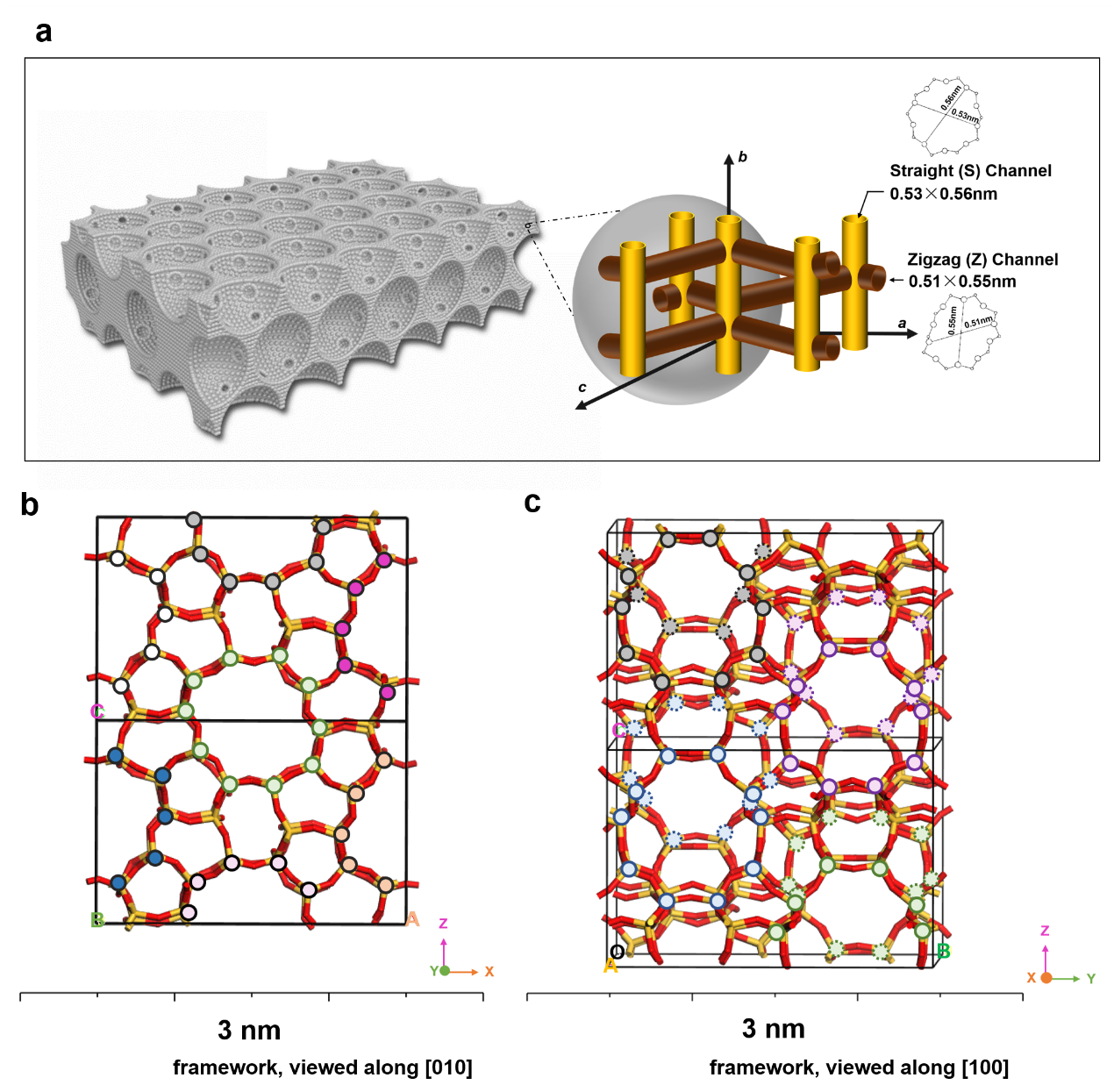


Figure S8 Schematic diagram of the micropores within one zeolite ZSM-5 nanocrystal. (a) The straight and zigzag channels within OMMM-ZSM-5 nanocrystals. (b) The straight channels along *b (010)* axis within a simulation cell consisting of two unit cells. Spheres with different colors refer to the silica atoms within the10-member rings belonging to different straight channels and (c) The zigzag channels along *a (100)* axis within a simulation cell consisting of two unit cells. Spheres with different colors refer to the silica atoms within the 10-member rings belonging to different zig-zag channels. For spheres with the same colors, the solid-edged spheres and dot-edged spheres refer to the silica atoms within two different 10-member rings belonging to the same zig-zag channels.

channels (Figure S3a). The straight channels along *b (010)* axis have pore openings defined by a cross-section of 10-member rings of 0.53×0.56 nm and zig-zag channels along *a (100)* axis by elliptic pores of 0.51×0.55 nm in cross-section axis.

To investigate the density distribution for the number of micropore channels, we choose a simulation cell consisting of two unit cells. The simulation cell has the dimensions of 2.00 nm× 1.97nm× (2×1.31) nm[6] and contains [1+6×(1/2)=4] straight channel through *XZ* plane (Figure S3b) and [2+2×(1/2)=3] zig-zag channels through *YZ* plane (Figure S3c).

Therefore, the density distribution for the average number of straight channels through *XZ* plane is 4/[2.00 nm×(2×1.31)nm]=0.76 nm^-2^. And the density distribution for the average number of zigzag channels through *YZ* plane is 3/[1.97 nm×(2×1.31)nm]=0.58 nm^-2^.

The average number of micropores (n) through the maximum cross-sectional area (along *Y* axis and *X* axis) in spherical zeolite ZSM-5 nanocrystals (diameter, d=20nm) was calculated as the following equation.

$\boldsymbol{n=}\frac{\boldsymbol{\pi}\boldsymbol{d}^{\boldsymbol{2}}}{\boldsymbol{4}}\boldsymbol{\times(0.76+0.58)}$ (Equation S4)

For OMMM-ZSM-5 zeolite, the average number of microprores within a spherical nanocrystal *n* is 421.


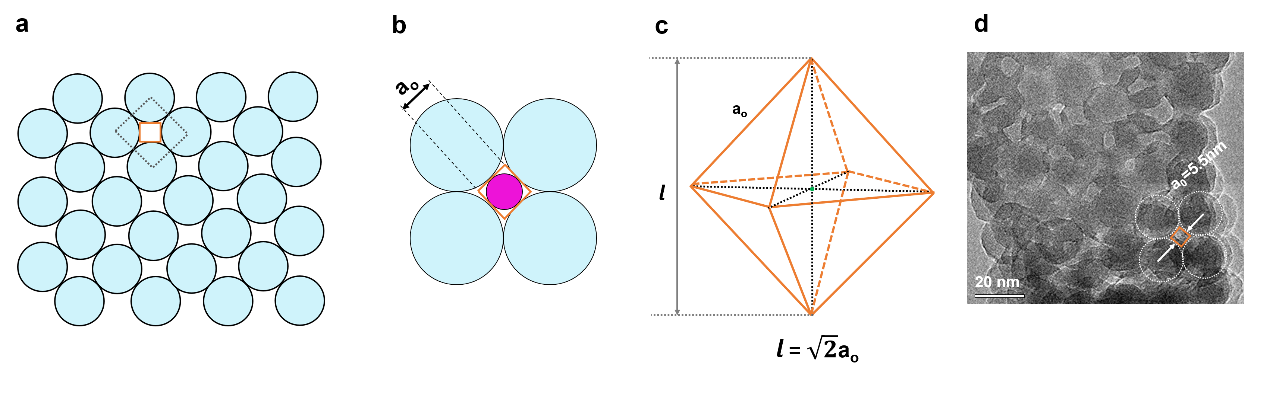


Figure S9 (a) Arrangement of spherical zeolite nanocrystals on the (100) plane of face-centered cubic (FCC) arrangement for OMMM-ZSM-5. (b) Four adjacent spherical zeolite nanocrystals around an octahedral void of the enlarged region in (a). (c) Illustration of the length *l* of the mesopores, equating to the average height of the octahedron surrounded by four adjacent zeolite nanocrystals. (d) SEM image of four adjacent spherical zeolite nanocrystals on the (100) plane.

For connecting mesopores to micropores, the exchange surface from mesopores and micropores and the mass variation *X* cannot be ignored. The *l* is the length of the mesopore, *i.e.*, the average height of the octahedron surrounded by four adjacent zeolite nanocrystals (Figure S4a, b and c) and thus equals to $\sqrt{2}$*a_0_*=$\sqrt{2}\times$5.5nm (Figure S4d). Based on the generalized Murray’s Law (Equation S2) when *D_micro_* = 0.5 nm, the optimized diameter of mesopores should thus be around 10 nm (Figure 2i). For our synthesized OMMM-ZSM-5, *D_meso_* = 8 nm, being very similar to the value predicted by the generalized Murray’s Law.

For connecting macropores to mesopores, the exchange surface from the macropores can be ignored relative to the whole exchange area of our macro-meso-microporous materials in chemical reactions. And the mass variation *X*=(*S_macro_*)/(*S_macro_*+*S_meso_*+*S_micro_*)<<1. According to the generalized Murray’s law (Equation S3), *h* is the diffusion distance from one macropore to another through meso-microporous wall.

Our OMMM-ZSM-5 zeolites were synthesized by using face-centered cubic (FCC) arranged colloidal crystal template formed by monodisperse polystyrene nanospheres with a close packed structure as the macropore template. In a close-packed FCC arranged polystyrene spheres, two kinds of interconnected voids exist: octahedral (*Oh*) and tetrahedral (*Td*). Figure S3 showed the *Oh* voids in OMMM-ZSM-5(400). These voids were filled with uniform zeolite nanocrystals. The *h* is the sum of *a_0_* (Figure S5a, b and c) and *2r_0_* (*D_macro_*) and thus equals to (*D_macro_*+120). Based on the generalized Murray’s Law (Equation S3) when *D_meso_* = 10 nm, the optimized diameters of macropores should be around 440 nm. For our synthesized OMMM-ZSM-5(400), *D_macro_* = 400 nm, which is approximatively equal to the value (440 nm) predicted by the generalized Murray’s Law. The above results clearly show that our OMMM-ZSM-5 fully obeys the generalized Murray’s Law and can be considered as hierarchical Murray zeolite.

Two more OMMM catalysts with different macropore sizes (200nm and 600nm) were made for a better comparison. Mesopores of 8 nm obtained (Figures S3d and S4d) meet quite well the size ratios between micro- and meso- pores. According to the generalized Murray’s Law (Equation S3, Figures S11 and S12), the macropore size should be 146 nm for OMMM-ZSM-5(200) and 812 nm for OMMM-ZSM-5(600). The macropore sizes of the obtained OMMM-ZSM-5(200) and OMMM-ZSM-5(600) are around 200nm and 600nm (Figure S3 and S4), which fails to match the theoretically predicted and optimized size ratios between macro- and meso- pores.


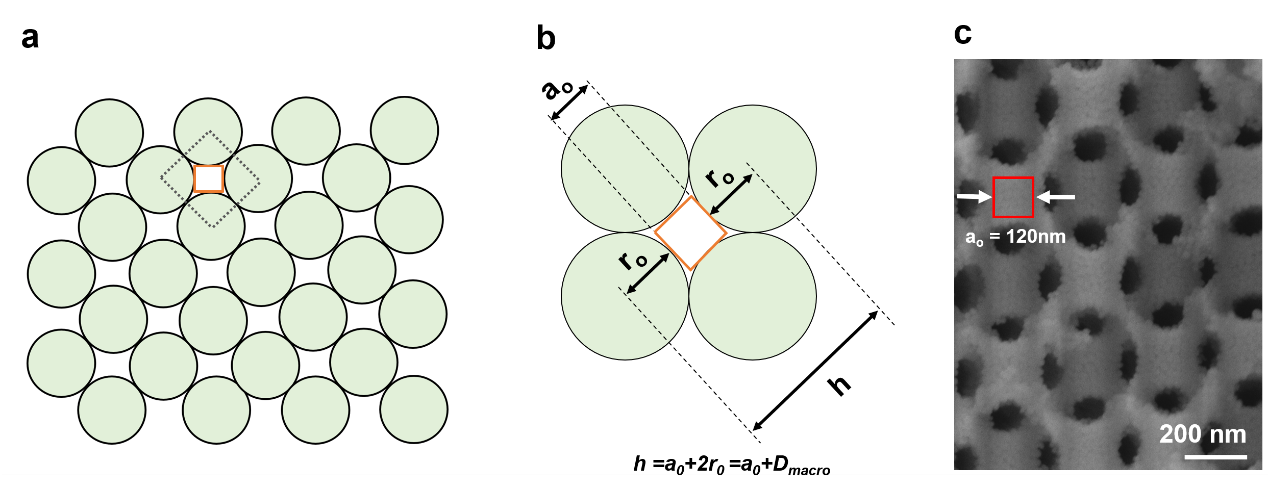


**Figure S10** (a) Arrangement of macroporous cages on the (100) plane of FCC arrangement for OMMM-ZSM-5(400). (b) Four adjacent macroporous cages around an octahedron of the enlarged region in (a). (c) SEM image of enlarged macroporous cages on the (100) plane.


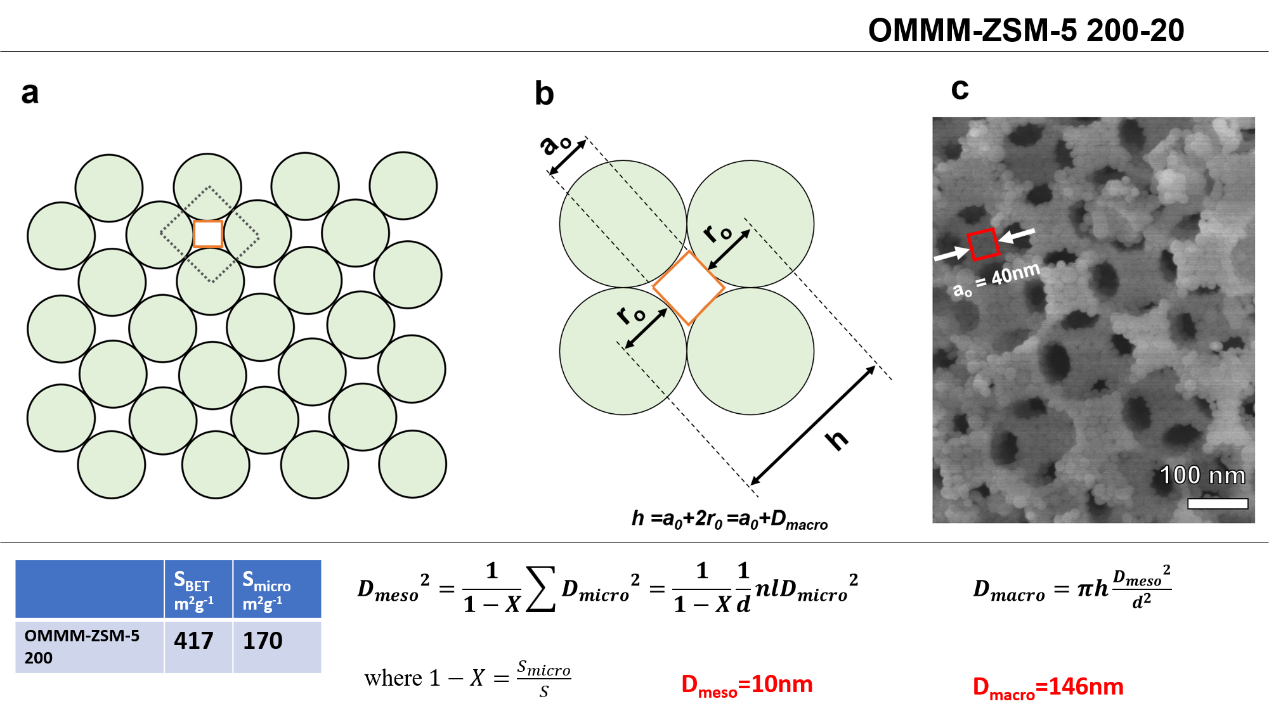


**Figure S11** (a) Arrangement of macroporous cages on the (100) plane of FCC arrangement for OMMM-ZSM-5(200). (b) Four adjacent macroporous cages around an octahedron of the enlarged region in (a). (c) SEM image of enlarged macroporous cages on the (100) plane.


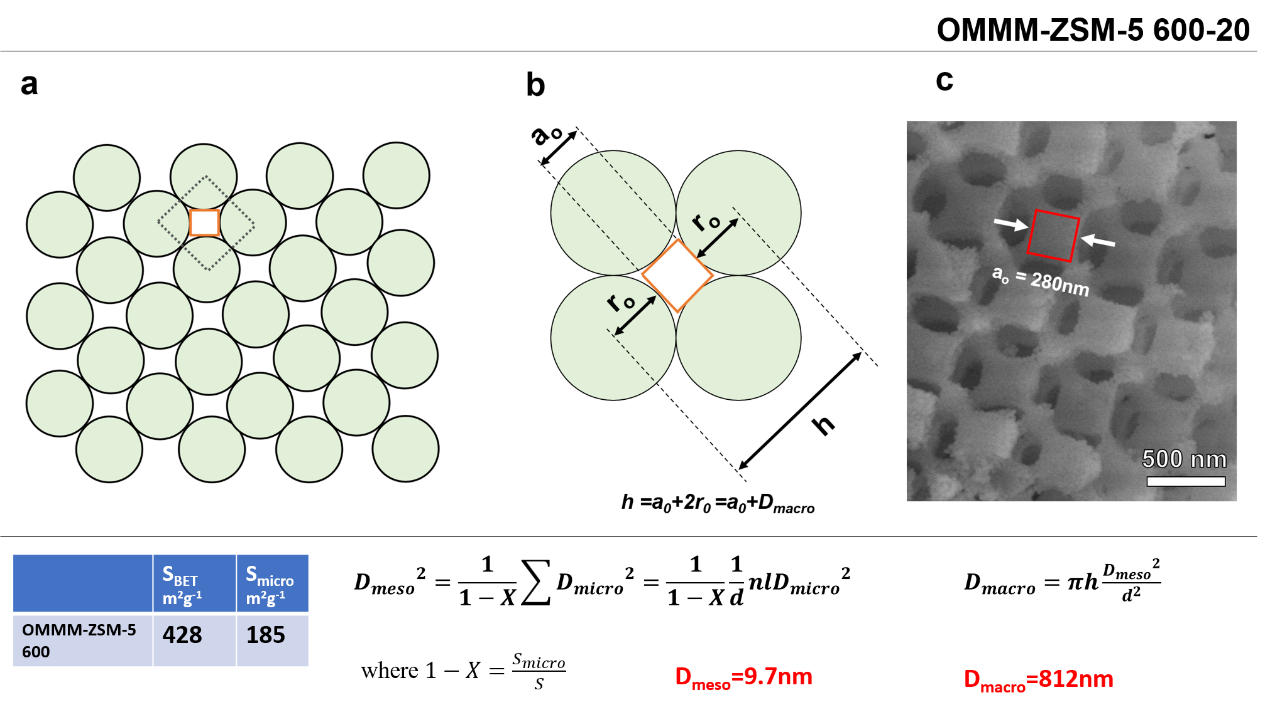


**Figure S12** (a) Arrangement of macroporous cages on the (100) plane of FCC arrangement for OMMM-ZSM-5(600). (b) Four adjacent macroporous cages around an octahedron of the enlarged region in (a). (c) SEM image of enlarged macroporous cages on the (100) plane.

**Diffusion studies**

***Laser hyperpolarized ^129^Xe NMR (HP ^129^Xe NMR)***

Laser hyperpolarized ^129^Xe NMR experiments were carried out at 110.6 MHz on a Varian Infinity-plus 400 spectrometer using a 7.5 mm probe. Before each experiment, samples (60-80 mesh) were dehydrated at 673 K under vacuum (<10^-5^ Torr) for 24 h. The optical polarization of xenon was achieved with a homemade apparatus with the optical pumping cell in the fringe field of the spectrometer magnet and a 60 W diode laser array (Coherent FAP-System). A flow of gas mixture (1% Xe-1% N_2_-98% He) was delivered at the rate of 100-150 mL min^-1^ to the sample in the detection region via plastic tubing. Variable-temperature NMR measurements were performed in the range of 153-273 K. All one-dimensional spectra were acquired with 3.0 μs π/2 pulse, 100-200 scans, and 2 s recycle delay. The chemical shifts were referenced to the signal of xenon gas. Although the line of the xenon gas is temperature dependent, generally chemical shifts vary no more than 1 ppm in the temperature range of the experiments.

***1,3,5-trimethylbenzene diffusion by a computer-controlled intelligent gravimetric analyzer (IGA)***

1,3,5-trimethylbenzene diffusion measurement in ZSM-5 zeolites was performed on a computer-controlled intelligent gravimetric analyzer (IGA 100B, Hiden Analytical Ltd., Warrington, UK). An ultrahigh vacuum system was employed by which adsorption isotherms and corresponding kinetics can be accurately acquired. A sensitive microbalance (resolution of 0.2 μg) was mounted in an enclosure with fitted thermostat to remove thermal coefficients of the weighing system and thus provide high stability and accuracy. The zeolite sample (100 ± 1 mg) was degassed under vacuum (<10^-5^ Pa) at 300 °C for 10h prior to the adsorption measurement. The system temperature was then set at 45 °C, regulated within 0.1 °C by a water bath. The relative pressure was rapidly raised to 0.099 and then kept constant during the tests. For each step, the amount of adsorbate introduced in the system was kept small enough to keep the adsorption process isothermal. The weight increase of sorbents was automatically measured by a microbalance and continuously recorded as a function of time under the settled gas vapor pressure.

Diffusion coefficient *D_c_* was obtained based on Fick’s Law. Equation S4 gives the fitting formula of diffusion coefficient *D_c_* for zeolite with geometry of flat plate.

$$\frac{Q_{t}-Q_{0}}{Q_{\infty}-Q_{0}}=1-\Sigma_{n=0}^{\infty}\frac{8}{\left[ \left( 2n+1 \right)\pi\right]^{2}}\exp\left[ \frac{-D_{c}(2n+1)^{2}\pi^{2}t}{h^{2}} \right]\approx\frac{8}{\sqrt{\pi^{2}}}{(\frac{D_{c}}{h^{2}})}^{1/2}\sqrt{t}$$

(when t is small) (Equation S5)

where Q_0_ is the initial adsorbed quantity, Q_t_ is the intermediate adsorbed quantity, Q_∞_ is the equilibrium adsorbed quantity, n is the natural numbers, *D_c_* is the diffusion coefficient for zeolite with geometry of flat plate, t is the adsorbed time, h is the thickness of zeolite.

***^1^H pulsed field gradient (PFG) NMR***

^1^H PFG NMR experiments were performed on a Bruker Avance III 600 MHz spectrometer equipped with a 5 mm diff50 diffusion probe which could provide a maximum gradient amplitude of 1800 G/cm in the Z-direction. A bipolar-gradient stimulated echo sequence (13-interval sequence) [7] was applied in diffusion tests in order to eliminate the effect of magnetic susceptibility in the beds of porous materials. Before PFG NMR test, a known amount of zeolite was dehydrated on a high vacuum system at 673 K for 12 h and then transferred into the NMR tube (Wilmad-LabGlass) with pressure valve in glove box. After degassing overnight at 393 K, a known amount of methane was quantitatively into NMR tube on a homemade uptake apparatus. Subsequently, the tube was sealed and equilibrated at room temperature. The loading of adsorbed methane was calculated by the ideal gas equation. All data were acquired at equilibrium condition.

Under the influence of field gradient pulses, the intensity M of the NMR signal (the ‘‘spin echo’’) is attenuated as expressed by the following the relation:

$\frac{\boldsymbol{M(\gamma\delta g,t)}}{\boldsymbol{M(0)}}\boldsymbol{\equiv\psi}\left( \boldsymbol{\gamma\delta g}\boldsymbol{,}\boldsymbol{t} \right)\boldsymbol{=}\int_{\boldsymbol{-\infty}}^{\boldsymbol{\infty}} \boldsymbol{P}\left( \boldsymbol{z}\boldsymbol{,}\boldsymbol{t} \right)\boldsymbol{cos}\left( \boldsymbol{\gamma\delta gz} \right)\boldsymbol{dz}$ (Equation S6)

where the term *γ**δg* represents the intensity of pulsed field gradients in which *δ* and *g* are the length and the amplitude of the pulses, respectively, and *γ* (=2.67×10^8^ T^-1^ s^-1^ for protons) denotes the gyromagnetic ratio of the nuclei under study[8]. *P(z,t)* (the mean propagator)[9] is the probability or density that an arbitrarily selected molecule within the sample is shifted over a distance *z* in a given direction (i.e. the applied field gradient) during the observation time *t* (the time interval between the two gradient pulses).

For molecular displacements in an isotropic medium, the molecular dynamics only contain one single parameter, that is, the coefficient of self-diffusion *D_f_*. In this case, the mean propagator is a simple Gaussian (Equation S7)

$\boldsymbol{P}\left( \boldsymbol{z,t} \right)\boldsymbol{=}\frac{\boldsymbol{1}}{\sqrt{\boldsymbol{4}\boldsymbol{\pi Dt}}}\mathbf{exp}\boldsymbol{(-}\frac{\boldsymbol{z(t)}^{\boldsymbol{2}}}{\boldsymbol{4}\boldsymbol{D}_{\boldsymbol{f}}\boldsymbol{t}}\boldsymbol{)}$ (Equation S7)

where *z(t)* is the mean-square displacement of the guest molecules in any arbitrary direction and obeys the Einstein relationship (Equation S8):

$\boldsymbol{<}\boldsymbol{z(t)}^{\boldsymbol{2}}\boldsymbol{> =2}\boldsymbol{D}_{\boldsymbol{f}}\boldsymbol{t}$ (Equation S8)

The mean squared value of the displacements *r(t)* of the molecules within the three-dimensional (3D) micropore system record during the observation time *t* is thus easily to be given by Equation S9:

$\boldsymbol{<}\boldsymbol{r}^{\boldsymbol{2}}\left( \boldsymbol{t} \right)\boldsymbol{>=6}\boldsymbol{D}_{\boldsymbol{f}}\boldsymbol{t}$ (Equation S9)

In the conditions where Equation S7 and Equation S8 are applicable, the molecules should have ‘normal diffusion’[10]. For normal diffusion, the PFG NMR signal attenuation is found to be given by the Equation (S5), which is obtained by Inserting Equation (S7) into Equation (S6).

$\boldsymbol{\psi}\left( \boldsymbol{\gamma\delta g}\boldsymbol{,}\boldsymbol{t} \right)\boldsymbol{=}\exp\left[ \boldsymbol{-}\left( \boldsymbol{\gamma\delta g} \right)^{\boldsymbol{2}}\boldsymbol{D}_{\boldsymbol{f}}\boldsymbol{t} \right]\boldsymbol{=}\boldsymbol{exp}\boldsymbol{[}\boldsymbol{-}\left( \boldsymbol{\gamma\delta g} \right)^{\boldsymbol{2}}\boldsymbol{<}\boldsymbol{z}^{\boldsymbol{2}}\boldsymbol{(t)>/2]}$ (Equation S10)

From Equation S10, the self-diffusivity *D_f_* is found to be the slope in a semi-logarithmic plot of the PFG NMR signal attenuation versus the squared field-gradient pulse intensity (*γδg*)^2^.

For Equation S9, only two limiting cases are considered, where mean diffusions path lengths are either much smaller or much larger than the sizes of the individual crystallites. Then the corresponding diffusivities are referred to as the coefficients of intracrystalline (*D_f-intra_*) and long-range (*D_f-long-range_*) diffusion.

For diffusion in porous materials[11-13], the above-mentioned two diffusivities existed. By considering the superposition of two expressions of the type given by Equations S5 and S6 to give Equations S11 and S12:

$$\psi\left( \gamma\delta g,t \right)=p_{long-range}(t)exp \left[ -\left( \gamma\delta g \right)^{2}D_{f-long-range}t \right]+p_{intra}(t)\exp\left[ -\left( \gamma\delta g \right)^{2}D_{f-intra}t \right]=p_{long-range}(t)exp[-\left( \gamma\delta g \right)^{2}\frac{{<z^{2}\left( t \right)>}_{long-range}}{2}+p_{intra}(t)exp[-\left( \gamma\delta g \right)^{2}\frac{{<z^{2}\left( t \right)>}_{intra}}{2}]$$

(Equation S11)

$$P\left( z,t \right)=p_{long-range}\frac{1}{\sqrt{4\pi D_{f-long-range}t}}\exp\left( -\frac{z^{2}}{4D_{f-long-range}t} \right)+p_{intra}\frac{1}{\sqrt{4\pi D_{f-intra}t}}exp(-\frac{z^{2}}{4D_{f-intra}t})$$

(Equation S12)

where the first term stands for diffusion and molecular exchange through the external space (long-range diffusion) and the second term denotes diffusion within the crystals (intracrystalline diffusion). The function *p_intra_(t)* stands for the relative number of molecules are still within their particles during the observation time *t* and the function *p_long-range_(t)*=1- *p_intra_(t)* for those that have exchanged between different particles during the observation time.


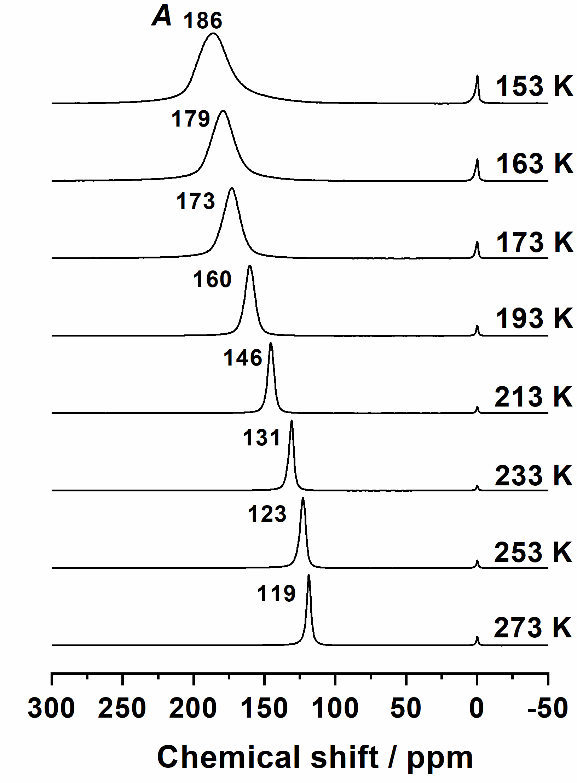


**Figure S13** Laser-hyperpolarized ^129^Xe NMR spectra with temperature varied from 273 K to 153 K of microporous ZSM-5.


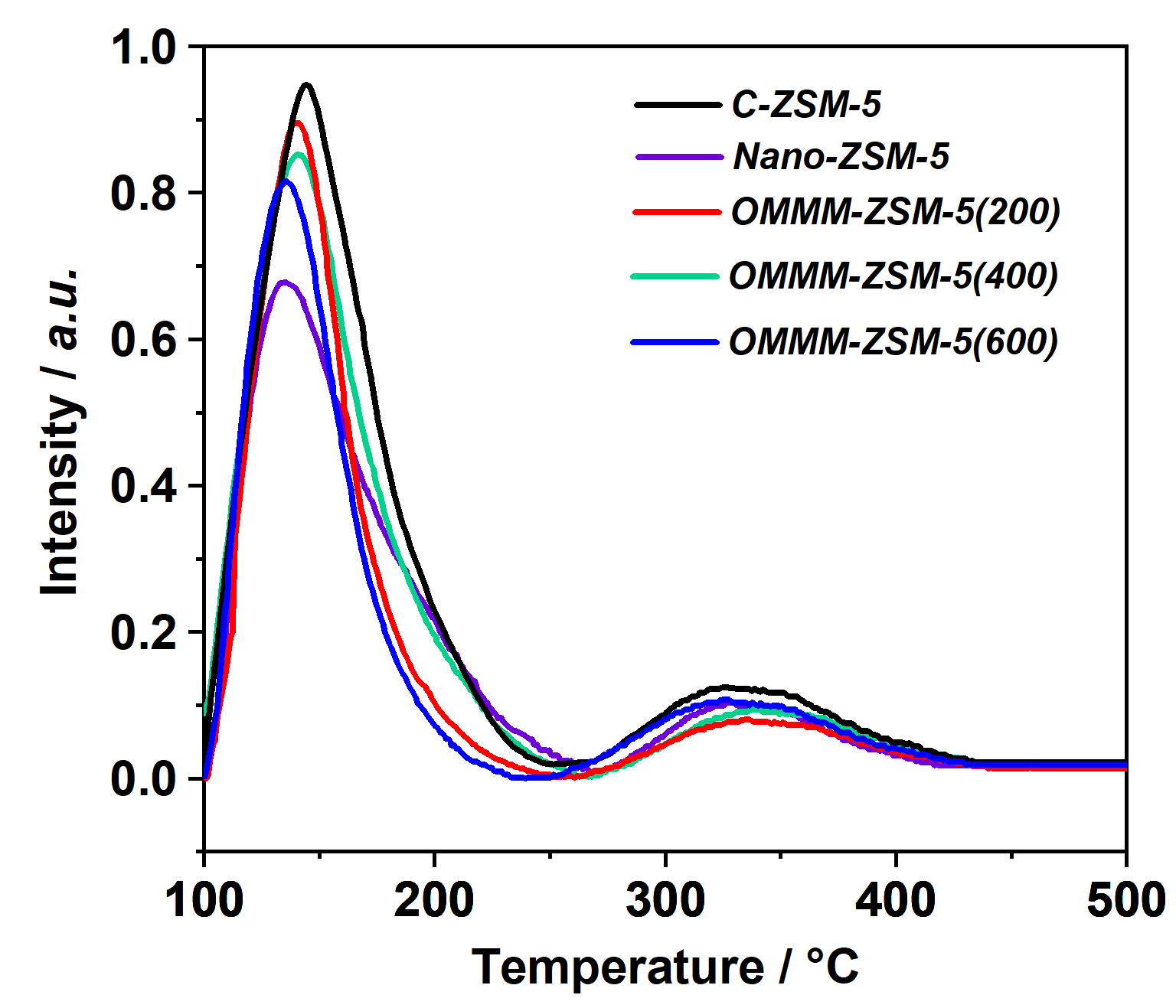


**Figure S14** NH_3_-TPD data of different ZSM-5 catalysts.

**Table S1 The structural parameters of various ZSM-5 catalysts**

| Catalyst | Surface area | | Pore volume | | |  |
| --- | --- | --- | --- | --- | --- | --- |
|  | S*_micro_*^a)^  [m^2^g^-1^] | S*_BET_* ^b)^ [m^2^g^-1^] | V*_mic_* ^c)^ [cm^3^g^-1^] | V*_total_* ^d)^ [cm^3^g^-1^] |  | |
| C-ZSM-5 | 153 | 220 | 0.08 | 0.15 |  | |
| Nano-ZSM-5 | 81 | 385 | 0.03 | 0.29 |  | |
| OMMM-ZSM-5(400)-0h | - | 166 | - | 0.17 |  | |
| OMMM-ZSM-5(400)-8h | 35 | 183 | 0.02 | 0.19 |  | |
| OMMM-ZSM-5(400)-16h | 62 | 224 | 0.03 | 0.22 |  | |
| OMMM-ZSM-5(400)-24h | 163 | 434 | 0.09 | 0.30 |  | |
| OMMM-ZSM-5(200) | 170 | 417 | 0.09 | 0.28 |  | |
| OMMM-ZSM-5(600) | 185 | 428 | 0.09 | 0.25 |  | |

^a)^ S*_micro_*: micropore surface area (m^2^g^-1^) determined from N_2_ adsorption using the *t*-plot method;

^b)^ S*_BET_*: BET Surface Area (m^2^g^-1^) determined from N_2_ adsorption-desorption isotherm;

^c)^ V*_micro_*: micropore volumn (cm^3^g^-1^) derived from N_2_ adsorption isotherm under relative pressure p/p_0_ of 0.01 using models based on non-local density functional theory (NLDFT);

^d)^ V*_total_*: Total pore volumn (cm^3^g^-1^) obtained from single point N_2_ adsorption total pore volume at P/P_0_ = 0.99;

**Table S2 Adsorption and diffusion properties of 1****,3,5-trimethybenzene over various ZSM-5 catalysts (40℃)**

| Sample | Max adsorption amount^a)^ (Q_max_ mg g^-1^) | Relative diffusion rate^b)^ (s^1/2^) |
| --- | --- | --- |
| C-ZSM-5 | 1.143 | 0.016 |
| Nano-ZSM-5 | 1.772 | 0.022 |
| OMMM-ZSM-5(200) | 8.874 | 0.093 |
| OMMM-ZSM-5(400) | 10.767 | 0.127 |
| OMMM-ZSM-5(600) | 10.175 | 0.0832 |

^a)^ Max adsorption amount was calculated from the trimethylbenzene when the relative pressure reaches up to 0.986.

^b)^ the slope of the fitted line in normalized uptake profile, in which t^1/2^ of the drops lower than 2 s^1/2^.

**Table S3** Acidic properties of the various zeolite ZSM-5 samples.

| Samples | Si/Al^a)^ | Total acidity ^b)^  [mmol NH_3_·  g^-1^ cat] | | Weak-acid peak^c)^  [mmol NH_3_·  g^-1^ cat] | Strong-acid peak^d)^  [mmol NH_3_·  g^-1^ cat] | Strong/Weak acid site ratio^e)^  [a.u.] | Total acidity ^f)^  [μmol TMPO·g^-1^ cat] | External surface acidity ^g)^  [μmol TBPO·g^-1^ cat] |
| --- | --- | --- | --- | --- | --- | --- | --- | --- |
| C-ZSM-5 | 52 | | 1.39 | 0.86 | 0.53 | 0.62 | 269 | 41 |
| Nano-ZSM-5 | 56 | | 1.30 | 0.85 | 0.45 | 0.53 | 253 | 79 |
| OMMM-ZSM-5(200) | 57 | | 1.27 | 0.81 | 0.46 | 0.57 | 251 | 119 |
| OMMM-ZSM-5(400) | 58 | | 1.25 | 0.78 | 0.47 | 0.60 | 248 | 124 |
| OMMM-ZSM-5(600) | 55 | | 1.31 | 0.83 | 0.48 | 0.58 | 255 | 131 |

^a)^Molar ratio in the solid, determined by ICP-AES.

^b)^Calculated from the amount of ammonia desorbed in the ammonia TPD analysis.

^c)^ Determined by the ammonia desorbed at 100℃-250℃ in the ammonia TPD analysis.

^d)^ Determined by the ammonia desorbed above 250℃ in the ammonia TPD analysis.

^e)^ Calculated by the ratio of LT-peak area/HT-peak in the ammonia TPD analysis.

^f)^Determined by the adsorption of TMPO.

^g)^Determined by the adsorption of TBPO.

**Table S4** Comparison of the catalytic selectivity at the similar conversion of 1,3,5-TIPB

| Catalysts | Conversion of 1,3,5-TIPB (%) | Product selectivity (%) | | | |
| --- | --- | --- | --- | --- | --- |
|  |  | DIPB | IPB | propylene | benzene |
| C-ZSM-5 | 32.4 | 58.1 | 8.9 | 26.7 | 4.3 |
| Nano- ZSM-5 | 32.1 | 36.2 | 14.2 | 35.4 | 13.1 |
| OMMM-ZSM-5(200) | 32.7 | 5.9 | 14.6 | 61.5 | 16.2 |
| OMMM-ZSM-5(400) | 33.1 | 4.1 | 9.3 | 64.3 | 20.3 |
| OMMM-ZSM-5(600) | 32.3 | 6.5 | 20.3 | 55.2 | 15.6 |

Reaction conditions: temperature = 280°C, WHSV_1,3,5-TIPB_ =1 h^-1^ (C-ZSM-5), WHSV_1,3,5-TIPB_ =1.4 h^-1^ (Nano-ZSM-5), WHSV_1,3,5-TIPB_ =4 h^-1^ [OMMM-ZSM-5(200)], WHSV_1,3,5-TIPB_ =4.5 h^-1^ [OMMM-ZSM-5(400)], WHSV_1,3,5-TIPB_ =4.5 h^-1^ [OMMM-ZSM-5(600)]. Time-on-stream = 4 h.

**Table S5** Comparison of catalyst performance in cracking of 1,3,5-TIPB

| Time on stream | | 1h | 2h | 3h | 4h | 5h | 6h | 7h | 8h |
| --- | --- | --- | --- | --- | --- | --- | --- | --- | --- |
| C-ZSM-5 | C_1,3,5-TIPB_ ^a^ /% | 25.7 | 22.4 | 16.8 | 12.7 | 8.9 | 6.8 | 4.9 | 2.7 |
|  | TON^b^ | 18.5 | 16.1 | 12.1 | 9.1 | 6.4 | 4.9 | 3.5 | 1.9 |
| Nano-  ZSM-5 | C_1,3,5-TIPB_ ^a^ /% | 48.4 | 41.4 | 37.8 | 32.8 | 29.9 | 25.8 | 20.9 | 16.7 |
|  | TON^b^ | 37.2 | 31.8 | 29.1 | 25.2 | 23.0 | 19.8 | 16.1 | 12.8 |
| OMMM-ZSM-5(200) | C_1,3,5-TIPB_ ^a^ /% | 57.3 | 54.8 | 53.3 | 51.7 | 50.9 | 48.8 | 47.6 | 46.2 |
|  | TON^b^ | 45.1 | 43.1 | 42.0 | 40.7 | 40.1 | 38.4 | 37.5 | 36.4 |
| OMMM-ZSM-5(400) | C_1,3,5-TIPB_ ^a^ /% | 62.7 | 61.8 | 60.4 | 58.9 | 57.2 | 55.3 | 54.2 | 52.6 |
|  | TON^b^ | 50.2 | 49.4 | 48.3 | 47.1 | 45.8 | 44.2 | 43.4 | 42.1 |
| OMMM-ZSM-5(600) | C_1,3,5-TIPB_ ^a^ /% | 64.1 | 63.2 | 61.9 | 60.2 | 58.5 | 56.5 | 55.2 | 53.8 |
|  | TON^b^ | 48.9 | 48.2 | 47.3 | 46.0 | 44.7 | 43.1 | 42.1 | 41.1 |

^a^The 1,3,5-TIPB catalytic cracking tests were performed at 300°C, atmosphere pressure, and a 1,3,5-TIPB WHSV of 3 h^–1^; 1,3,5-TIPB conversion and product distribution were determined at periodically on stream.

^b^The turnover number (TON) is the accumulated number of 1,3,5-TIPB molecules converted at time on stream of *t* per acidity. TON = Moles of desired product formed per acidity (total acidity measured by NH_3_-TPD analysis)

**Table S6** The coke deposition on the reacted catalysts

| Samples | C-ZSM-5 | Nano-ZSM-5 | OMMM-  ZSM-5(200) | OMMM-  ZSM-5(400) | OMMM-  ZSM-5(400) |
| --- | --- | --- | --- | --- | --- |
| Weigh loss /% | 13.3 | 9.4 | 7.8 | 6.7 | 6.4 |

^a^The coke deposition on the reacted samples was analyzed by the thermogravimetric analysis of catalysts used in cracking of 1,3,5-TIPB for 8h.

**Reference**

1. Zhang S, Chen L and Zhou S *et al.* Facile Synthesis of Hierarchically Ordered Porous Carbon via in Situ Self-Assembly of Colloidal Polymer and Silica Spheres and Its Use as a Catalyst Support. *Chem Mater* 2010; **22**: 3433-40.

2. Zhao Q, Chen WH and Huang SJ *et al.* Discernment and quantification of internal and external acid sites on zeolites. *J Phys Chem B* 2002; **106**: 4462-69.

3. Zhu J, Zhu Y and Zhu L *et al.* Highly mesoporous single-crystalline zeolite beta synthesized using a nonsurfactant cationic polymer as a dual-function template. *J Am Chem Soc* 2014; **136**: 2503-10.

4. Travkina OS, Agliullin MR and Filippova NA *et al.* Template-free synthesis of high degree crystallinity zeolite Y with micro–meso–macroporous structure. *RSC Adv* 2017; **7**: 32581-90.

5. Zheng X, Shen G and Wang C *et al.* Bio-inspired murray materials for mass transfer and activity. *Nat Commun* 2017; **8**: 14921.

6. Kokotailo GT, Lawton SL and Olson DH *et al.* Structure of synthetic zeolite ZSM-5. *Nature* 1978; **272**: 437-8.

7. Cotts RM, Hoch MJR and Sun T *et al.* Pulsed field gradient stimulated echo methods for improved NMR diffusion measurements in heterogeneous systems. *Journal of Magnetic Resonance (1969-1992)* 1989; **83**: 252-66.

8. Mehlhorn D, Inayat A and Schwieger W *et al.* Probing mass transfer in mesoporous Faujasite-type zeolite nanosheet assemblies. *ChemPhysChem* 2014; **15**: 1681-6.

9. Kärger J and Heink W. The propagator representation of molecular transport in microporous crystallites. *J Magn Reson* 1983; **51**: 1-7.

10. Kärger J and Ruthven DM. Diffusion in nanoporous materials: fundamental principles, insights and challenges. *New J Chem* 2016; **40**: 4027-48.

11. Kärger J and Valiullin R. Mass transfer in mesoporous materials: the benefit of microscopic diffusion measurement. *Chem Soc Rev* 2013; **42**: 4172-4197.

12. Mehlhorn D, Valiullin R and Kärger J *et al.* Exploring the hierarchy of transport phenomena in hierarchical pore systems by NMR diffusion measurement. *Microporous and Mesoporous Materials* 2012; **164**: 273-9.

13. Mehlhorn D, Valiullin R and Kärger J *et al.* Intracrystalline diffusion in mesoporous zeolites. *ChemPhysChem* 2012; **13**: 1495-9.
